# Event generator tunes obtained from underlying event and multiparton scattering measurements

**DOI:** 10.1140/epjc/s10052-016-3988-x

**Published:** 2016-03-17

**Authors:** V. Khachatryan, A. M. Sirunyan, A. Tumasyan, W. Adam, E. Asilar, T. Bergauer, J. Brandstetter, E. Brondolin, M. Dragicevic, J. Erö, M. Friedl, R. Frühwirth, V. M. Ghete, C. Hartl, N. Hörmann, J. Hrubec, M. Jeitler, V. Knünz, A. König, M. Krammer, I. Krätschmer, D. Liko, T. Matsushita, I. Mikulec, D. Rabady, B. Rahbaran, H. Rohringer, J. Schieck, R. Schöfbeck, J. Strauss, W. Treberer-Treberspurg, W. Waltenberger, C.-E. Wulz, V. Mossolov, N. Shumeiko, J. Suarez Gonzalez, S. Alderweireldt, T. Cornelis, E. A. De Wolf, X. Janssen, A. Knutsson, J. Lauwers, S. Luyckx, M. Van De Klundert, H. Van Haevermaet, P. Van Mechelen, N. Van Remortel, A. Van Spilbeeck, S. Abu Zeid, F. Blekman, J. D’Hondt, N. Daci, I. De Bruyn, K. Deroover, N. Heracleous, J. Keaveney, S. Lowette, L. Moreels, A. Olbrechts, Q. Python, D. Strom, S. Tavernier, W. Van Doninck, P. Van Mulders, G. P. Van Onsem, I. Van Parijs, P. Barria, H. Brun, C. Caillol, B. Clerbaux, G. De Lentdecker, G. Fasanella, L. Favart, A. Grebenyuk, G. Karapostoli, T. Lenzi, A. Léonard, T. Maerschalk, A. Marinov, L. Perniè, A. Randle-conde, T. Seva, C. Vander Velde, R. Yonamine, P. Vanlaer, R. Yonamine, F. Zenoni, F. Zhang, V. Adler, K. Beernaert, L. Benucci, A. Cimmino, S. Crucy, D. Dobur, A. Fagot, G. Garcia, M. Gul, J. Mccartin, A. A. Ocampo Rios, D. Poyraz, D. Ryckbosch, S. Salva, M. Sigamani, M. Tytgat, W. Van Driessche, E. Yazgan, N. Zaganidis, S. Basegmez, C. Beluffi, O. Bondu, S. Brochet, G. Bruno, A. Caudron, L. Ceard, G. G. Da Silveira, C. Delaere, D. Favart, L. Forthomme, A. Giammanco, J. Hollar, A. Jafari, P. Jez, M. Komm, V. Lemaitre, A. Mertens, M. Musich, C. Nuttens, L. Perrini, A. Pin, K. Piotrzkowski, A. Popov, L. Quertenmont, M. Selvaggi, M. Vidal Marono, N. Beliy, G. H. Hammad, W. L. Aldá Júnior, F. L. Alves, G. A. Alves, L. Brito, M. Correa Martins Junior, M. Hamer, C. Hensel, A. Moraes, M. E. Pol, P. Rebello Teles, E. Belchior Batista Das Chagas, W. Carvalho, J. Chinellato, A. Custódio, E. M. Da Costa, D. De Jesus Damiao, C. De Oliveira Martins, S. Fonseca De Souza, L. M. Huertas Guativa, H. Malbouisson, D. Matos Figueiredo, C. Mora Herrera, L. Mundim, H. Nogima, W. L. Prado Da Silva, A. Santoro, A. Sznajder, E. J. Tonelli Manganote, A. Vilela Pereira, S. Ahuja, C. A. Bernardes, A. De Souza Santos, S. Dogra, T. R. Fernandez Perez Tomei, E. M. Gregores, P. G. Mercadante, C. S. Moon, S. F. Novaes, Sandra S. Padula, D. Romero Abad, J. C. Ruiz Vargas, A. Aleksandrov, R. Hadjiiska, P. Iaydjiev, M. Rodozov, S. Stoykova, G. Sultanov, M. Vutova, A. Dimitrov, I. Glushkov, L. Litov, B. Pavlov, P. Petkov, M. Ahmad, J. G. Bian, G. M. Chen, H. S. Chen, M. Chen, T. Cheng, R. Du, C. H. Jiang, R. Plestina, F. Romeo, S. M. Shaheen, A. Spiezia, J. Tao, C. Wang, Z. Wang, H. Zhang, C. Asawatangtrakuldee, Y. Ban, Q. Li, S. Liu, Y. Mao, S. J. Qian, D. Wang, Z. Xu, C. Avila, A. Cabrera, L. F. Chaparro Sierra, C. Florez, J. P. Gomez, B. Gomez Moreno, J. C. Sanabria, N. Godinovic, D. Lelas, I. Puljak, P. M. Ribeiro Cipriano, Z. Antunovic, M. Kovac, V. Brigljevic, K. Kadija, J. Luetic, S. Micanovic, L. Sudic, A. Attikis, G. Mavromanolakis, J. Mousa, C. Nicolaou, F. Ptochos, P. A. Razis, H. Rykaczewski, M. Bodlak, M. Finger, M. Finger, A. A. Abdelalim, A. Awad, A. Mahrous, Y. Mohammed, A. Radi, B. Calpas, M. Kadastik, M. Murumaa, M. Raidal, A. Tiko, C. Veelken, P. Eerola, J. Pekkanen, M. Voutilainen, J. Härkönen, V. Karimäki, R. Kinnunen, T. Lampén, K. Lassila-Perini, S. Lehti, T. Lindén, P. Luukka, T. Mäenpää, T. Peltola, E. Tuominen, J. Tuominiemi, E. Tuovinen, L. Wendland, J. Talvitie, T. Tuuva, M. Besancon, F. Couderc, M. Dejardin, D. Denegri, B. Fabbro, J. L. Faure, C. Favaro, F. Ferri, S. Ganjour, A. Givernaud, P. Gras, G. Hamel de Monchenault, P. Jarry, E. Locci, M. Machet, J. Malcles, J. Rander, A. Rosowsky, M. Titov, A. Zghiche, I. Antropov, S. Baffioni, F. Beaudette, P. Busson, L. Cadamuro, E. Chapon, C. Charlot, T. Dahms, O. Davignon, N. Filipovic, R. Granier de Cassagnac, M. Jo, S. Lisniak, L. Mastrolorenzo, P. Miné, I. N. Naranjo, M. Nguyen, C. Ochando, G. Ortona, P. Paganini, P. Pigard, S. Regnard, R. Salerno, J. B. Sauvan, Y. Sirois, T. Strebler, Y. Yilmaz, A. Zabi, J.-L. Agram, J. Andrea, A. Aubin, D. Bloch, J.-M. Brom, M. Buttignol, E. C. Chabert, N. Chanon, C. Collard, E. Conte, X. Coubez, J.-C. Fontaine, D. Gelé, U. Goerlach, C. Goetzmann, A.-C. Le Bihan, J. A. Merlin, K. Skovpen, P. Van Hove, S. Gadrat, S. Beauceron, C. Bernet, G. Boudoul, E. Bouvier, C. A. Carrillo Montoya, R. Chierici, D. Contardo, B. Courbon, P. Depasse, H. El Mamouni, J. Fan, J. Fay, S. Gascon, M. Gouzevitch, B. Ille, F. Lagarde, I. B. Laktineh, M. Lethuillier, L. Mirabito, A. L. Pequegnot, S. Perries, J. D. Ruiz Alvarez, D. Sabes, L. Sgandurra, V. Sordini, M. Vander Donckt, P. Verdier, S. Viret, T. Toriashvili, D. Lomidze, C. Autermann, S. Beranek, M. Edelhoff, L. Feld, A. Heister, M. K. Kiesel, K. Klein, M. Lipinski, A. Ostapchuk, M. Preuten, F. Raupach, S. Schael, J. F. Schulte, T. Verlage, H. Weber, B. Wittmer, V. Zhukov, M. Ata, M. Brodski, E. Dietz-Laursonn, D. Duchardt, M. Endres, M. Erdmann, S. Erdweg, T. Esch, R. Fischer, A. Güth, T. Hebbeker, C. Heidemann, K. Hoepfner, S. Knutzen, P. Kreuzer, M. Merschmeyer, A. Meyer, P. Millet, M. Olschewski, K. Padeken, P. Papacz, T. Pook, M. Radziej, H. Reithler, M. Rieger, F. Scheuch, L. Sonnenschein, D. Teyssier, S. Thüer, V. Cherepanov, Y. Erdogan, G. Flügge, H. Geenen, M. Geisler, F. Hoehle, B. Kargoll, T. Kress, Y. Kuessel, A. Künsken, J. Lingemann, A. Nehrkorn, A. Nowack, I. M. Nugent, C. Pistone, O. Pooth, A. Stahl, M. Aldaya Martin, I. Asin, N. Bartosik, O. Behnke, U. Behrens, A. J. Bell, K. Borras, A. Burgmeier, A. Campbell, S. Choudhury, F. Costanza, C. Diez Pardos, G. Dolinska, S. Dooling, T. Dorland, G. Eckerlin, D. Eckstein, T. Eichhorn, G. Flucke, E. Gallo, J. Garay Garcia, A. Geiser, A. Gizhko, P. Gunnellini, J. Hauk, M. Hempel, H. Jung, A. Kalogeropoulos, O. Karacheban, M. Kasemann, P. Katsas, J. Kieseler, C. Kleinwort, I. Korol, W. Lange, J. Leonard, K. Lipka, A. Lobanov, W. Lohmann, R. Mankel, I. Marfin, I.-A. Melzer-Pellmann, A. B. Meyer, G. Mittag, J. Mnich, A. Mussgiller, S. Naumann-Emme, A. Nayak, E. Ntomari, H. Perrey, D. Pitzl, R. Placakyte, A. Raspereza, B. Roland, M. Ö. Sahin, P. Saxena, T. Schoerner-Sadenius, M. Schröder, C. Seitz, S. Spannagel, K. D. Trippkewitz, R. Walsh, C. Wissing, V. Blobel, M. Centis Vignali, A. R. Draeger, J. Erfle, E. Garutti, K. Goebel, D. Gonzalez, M. Görner, J. Haller, M. Hoffmann, R. S. Höing, A. Junkes, R. Klanner, R. Kogler, N. Kovalchuk, T. Lapsien, T. Lenz, I. Marchesini, D. Marconi, M. Meyer, D. Nowatschin, J. Ott, F. Pantaleo, T. Peiffer, A. Perieanu, N. Pietsch, J. Poehlsen, D. Rathjens, C. Sander, C. Scharf, H. Schettler, P. Schleper, E. Schlieckau, A. Schmidt, J. Schwandt, V. Sola, H. Stadie, G. Steinbrück, H. Tholen, D. Troendle, E. Usai, L. Vanelderen, A. Vanhoefer, B. Vormwald, C. Barth, C. Baus, J. Berger, C. Böser, E. Butz, T. Chwalek, F. Colombo, W. De Boer, A. Descroix, A. Dierlamm, S. Fink, F. Frensch, R. Friese, M. Giffels, A. Gilbert, D. Haitz, F. Hartmann, S. M. Heindl, U. Husemann, I. Katkov, A. Kornmayer, P. Lobelle Pardo, B. Maier, H. Mildner, M. U. Mozer, T. Müller, Th. Müller, M. Plagge, G. Quast, K. Rabbertz, S. Röcker, F. Roscher, G. Sieber, H. J. Simonis, F. M. Stober, R. Ulrich, J. Wagner-Kuhr, S. Wayand, M. Weber, T. Weiler, S. Williamson, C. Wöhrmann, R. Wolf, G. Anagnostou, G. Daskalakis, T. Geralis, V. A. Giakoumopoulou, A. Kyriakis, D. Loukas, A. Psallidas, I. Topsis-Giotis, A. Agapitos, S. Kesisoglou, A. Panagiotou, N. Saoulidou, E. Tziaferi, I. Evangelou, G. Flouris, C. Foudas, P. Kokkas, N. Loukas, N. Manthos, I. Papadopoulos, E. Paradas, J. Strologas, G. Bencze, C. Hajdu, A. Hazi, P. Hidas, D. Horvath, F. Sikler, V. Veszpremi, G. Vesztergombi, A. J. Zsigmond, N. Beni, S. Czellar, J. Karancsi, J. Molnar, Z. Szillasi, M. Bartók, A. Makovec, P. Raics, Z. L. Trocsanyi, B. Ujvari, P. Mal, K. Mandal, D. K. Sahoo, N. Sahoo, S. K. Swain, S. Bansal, S. B. Beri, V. Bhatnagar, R. Chawla, R. Gupta, U. Bhawandeep, A. K. Kalsi, A. Kaur, M. Kaur, R. Kumar, A. Mehta, M. Mittal, J. B. Singh, G. Walia, Ashok Kumar, A. Bhardwaj, B. C. Choudhary, R. B. Garg, A. Kumar, S. Malhotra, M. Naimuddin, N. Nishu, K. Ranjan, R. Sharma, V. Sharma, S. Bhattacharya, K. Chatterjee, S. Dey, S. Dutta, Sa. Jain, N. Majumdar, A. Modak, K. Mondal, S. Mukherjee, S. Mukhopadhyay, A. Roy, D. Roy, S. Roy Chowdhury, S. Sarkar, M. Sharan, A. Abdulsalam, R. Chudasama, D. Dutta, V. Jha, V. Kumar, A. K. Mohanty, L. M. Pant, P. Shukla, A. Topkar, T. Aziz, S. Banerjee, S. Bhowmik, R. M. Chatterjee, R. K. Dewanjee, S. Dugad, S. Ganguly, S. Ghosh, M. Guchait, A. Gurtu, G. Kole, S. Kumar, B. Mahakud, M. Maity, G. Majumder, K. Mazumdar, S. Mitra, G. B. Mohanty, B. Parida, T. Sarkar, N. Sur, B. Sutar, N. Wickramage, S. Chauhan, S. Dube, A. Kapoor, K. Kothekar, S. Sharma, H. Bakhshiansohi, H. Behnamian, S. M. Etesami, A. Fahim, R. Goldouzian, M. Khakzad, M. Mohammadi Najafabadi, M. Naseri, S. Paktinat Mehdiabadi, F. Rezaei Hosseinabadi, B. Safarzadeh, M. Zeinali, M. Felcini, M. Grunewald, M. Abbrescia, C. Calabria, C. Caputo, A. Colaleo, D. Creanza, L. Cristella, N. De Filippis, M. De Palma, L. Fiore, G. Iaselli, G. Maggi, G. Miniello, M. Maggi, S. My, S. Nuzzo, A. Pompili, G. Pugliese, R. Radogna, A. Ranieri, G. Selvaggi, L. Silvestris, R. Venditti, P. Verwilligen, G. Abbiendi, C. Battilana, A. C. Benvenuti, D. Bonacorsi, S. Braibant-Giacomelli, L. Brigliadori, R. Campanini, P. Capiluppi, A. Castro, F. R. Cavallo, S. S. Chhibra, G. Codispoti, M. Cuffiani, G. M. Dallavalle, F. Fabbri, A. Fanfani, D. Fasanella, P. Giacomelli, C. Grandi, L. Guiducci, S. Marcellini, G. Masetti, A. Montanari, F. L. Navarria, A. Perrotta, A. M. Rossi, F. Primavera, T. Rovelli, G. P. Siroli, N. Tosi, R. Travaglini, G. Cappello, M. Chiorboli, S. Costa, A. Di Mattia, F. Giordano, R. Potenza, A. Tricomi, C. Tuve, G. Barbagli, V. Ciulli, C. Civinini, R. D’Alessandro, E. Focardi, S. Gonzi, V. Gori, P. Lenzi, M. Meschini, S. Paoletti, G. Sguazzoni, A. Tropiano, L. Viliani, L. Benussi, S. Bianco, F. Fabbri, D. Piccolo, F. Primavera, V. Calvelli, F. Ferro, M. Lo Vetere, M. R. Monge, E. Robutti, S. Tosi, L. Brianza, M. E. Dinardo, S. Fiorendi, S. Gennai, R. Gerosa, A. Ghezzi, P. Govoni, S. Malvezzi, R. A. Manzoni, B. Marzocchi, D. Menasce, L. Moroni, M. Paganoni, D. Pedrini, S. Ragazzi, N. Redaelli, T. Tabarelli de Fatis, S. Buontempo, N. Cavallo, S. Di Guida, M. Esposito, F. Fabozzi, A. O. M. Iorio, G. Lanza, L. Lista, S. Meola, M. Merola, P. Paolucci, C. Sciacca, F. Thyssen, P. Azzi, N. Bacchetta, L. Benato, D. Bisello, A. Boletti, A. Branca, R. Carlin, P. Checchia, M. Dall’Osso, T. Dorigo, U. Dosselli, S. Fantinel, F. Fanzago, F. Gasparini, U. Gasparini, A. Gozzelino, K. Kanishchev, S. Lacaprara, M. Margoni, A. T. Meneguzzo, J. Pazzini, N. Pozzobon, P. Ronchese, F. Simonetto, E. Torassa, M. Tosi, M. Zanetti, P. Zotto, A. Zucchetta, A. Braghieri, A. Magnani, P. Montagna, S. P. Ratti, V. Re, C. Riccardi, P. Salvini, I. Vai, P. Vitulo, L. Alunni Solestizi, G. M. Bilei, D. Ciangottini, L. Fanò, P. Lariccia, G. Mantovani, M. Menichelli, A. Saha, A. Santocchia, K. Androsov, P. Azzurri, G. Bagliesi, J. Bernardini, T. Boccali, R. Castaldi, M. A. Ciocci, R. Dell’Orso, S. Donato, G. Fedi, F. Fiori, L. Foà, A. Giassi, M. T. Grippo, F. Ligabue, T. Lomtadze, L. Martini, A. Messineo, F. Palla, A. Rizzi, A. Savoy-Navarro, A. T. Serban, P. Spagnolo, R. Tenchini, G. Tonelli, A. Venturi, P. G. Verdini, L. Barone, F. Cavallari, G. D’imperio, D. Del Re, M. Diemoz, S. Gelli, C. Jorda, E. Longo, F. Margaroli, P. Meridiani, G. Organtini, R. Paramatti, F. Preiato, S. Rahatlou, C. Rovelli, F. Santanastasio, P. Traczyk, N. Amapane, R. Arcidiacono, S. Argiro, M. Arneodo, R. Bellan, C. Biino, N. Cartiglia, M. Costa, R. Covarelli, A. Degano, N. Demaria, L. Finco, B. Kiani, C. Mariotti, S. Maselli, E. Migliore, V. Monaco, E. Monteil, M. M. Obertino, L. Pacher, N. Pastrone, M. Pelliccioni, G. L. Pinna Angioni, F. Ravera, A. Potenza, A. Romero, M. Ruspa, R. Sacchi, A. Solano, A. Staiano, S. Belforte, V. Candelise, M. Casarsa, F. Cossutti, G. Della Ricca, B. Gobbo, C. La Licata, M. Marone, A. Schizzi, A. Zanetti, T. A. Kropivnitskaya, S. K. Nam, D. H. Kim, G. N. Kim, M. S. Kim, M. S. Kim, D. J. Kong, S. Lee, Y. D. Oh, A. Sakharov, D. C. Son, J. A. Brochero Cifuentes, H. Kim, T. J. Kim, S. Song, S. Choi, Y. Go, D. Gyun, B. Hong, H. Kim, Y. Kim, B. Lee, K. Lee, K. S. Lee, S. Lee, S. Lee, S. K. Park, Y. Roh, H. D. Yoo, M. Choi, H. Kim, J. H. Kim, J. S. H. Lee, I. C. Park, G. Ryu, M. S. Ryu, Y. Choi, J. Goh, D. Kim, E. Kwon, J. Lee, I. Yu, V. Dudenas, A. Juodagalvis, J. Vaitkus, I. Ahmed, Z. A. Ibrahim, J. R. Komaragiri, M. A. B. Md Ali, F. Mohamad Idris, W. A. T. Wan Abdullah, M. N. Yusli, W. A. T. Wan Abdullah, E. Casimiro Linares, H. Castilla-Valdez, E. De La Cruz-Burelo, I. Heredia-De La Cruz, A. Hernandez-Almada, R. Lopez-Fernandez, A. Sanchez-Hernandez, S. Carrillo Moreno, F. Vazquez Valencia, I. Pedraza, H. A. Salazar Ibarguen, A. Morelos Pineda, D. Krofcheck, P. H. Butler, A. Ahmad, M. Ahmad, Q. Hassan, H. R. Hoorani, W. A. Khan, T. Khurshid, M. Shoaib, H. Bialkowska, M. Bluj, B. Boimska, T. Frueboes, M. Górski, M. Kazana, K. Nawrocki, K. Romanowska-Rybinska, M. Szleper, P. Zalewski, G. Brona, K. Bunkowski, A. Byszuk, K. Doroba, A. Kalinowski, M. Konecki, J. Krolikowski, M. Misiura, M. Olszewski, M. Walczak, P. Bargassa, C. Beir ao Da Cruz E Silva, A. Di Francesco, P. Faccioli, P. G. Ferreira Parracho, M. Gallinaro, N. Leonardo, L. Lloret Iglesias, F. Nguyen, J. Rodrigues Antunes, J. Seixas, O. Toldaiev, D. Vadruccio, J. Varela, P. Vischia, S. Afanasiev, P. Bunin, M. Gavrilenko, I. Golutvin, I. Gorbunov, A. Kamenev, V. Karjavin, V. Konoplyanikov, A. Lanev, A. Malakhov, V. Matveev, P. Moisenz, V. Palichik, V. Perelygin, M. Savina, S. Shmatov, S. Shulha, V. Smirnov, A. Zarubin, V. Golovtsov, Y. Ivanov, V. Kim, E. Kuznetsova, P. Levchenko, V. Murzin, V. Oreshkin, I. Smirnov, V. Sulimov, L. Uvarov, S. Vavilov, A. Vorobyev, Yu. Andreev, A. Dermenev, S. Gninenko, N. Golubev, A. Karneyeu, M. Kirsanov, N. Krasnikov, A. Pashenkov, D. Tlisov, A. Toropin, V. Epshteyn, V. Gavrilov, N. Lychkovskaya, V. Popov, l. Pozdnyakov, G. Safronov, A. Spiridonov, E. Vlasov, A. Zhokin, A. Bylinkin, V. Andreev, M. Azarkin, I. Dremin, M. Kirakosyan, A. Leonidov, G. Mesyats, S. V. Rusakov, A. Baskakov, A. Belyaev, E. Boos, M. Dubinin, L. Dudko, A. Ershov, A. Gribushin, V. Klyukhin, O. Kodolova, I. Lokhtin, I. Myagkov, S. Obraztsov, S. Petrushanko, V. Savrin, A. Snigirev, I. Azhgirey, I. Bayshev, S. Bitioukov, V. Kachanov, A. Kalinin, D. Konstantinov, V. Krychkine, V. Petrov, R. Ryutin, A. Sobol, L. Tourtchanovitch, S. Troshin, N. Tyurin, A. Uzunian, A. Volkov, P. Adzic, P. Cirkovic, J. Milosevic, V. Rekovic, J. Alcaraz Maestre, C. Battilana, E. Calvo, M. Cerrada, M. Chamizo Llatas, N. Colino, B. De La Cruz, A. Delgado Peris, A. Escalante Del Valle, C. Fernandez Bedoya, J. P. Fernández Ramos, J. Flix, M. C. Fouz, P. Garcia-Abia, O. Gonzalez Lopez, S. Goy Lopez, J. M. Hernandez, M. I. Josa, E. Navarro De Martino, A. Pérez-Calero Yzquierdo, J. Puerta Pelayo, A. Quintario Olmeda, I. Redondo, L. Romero, J. Santaolalla, M. S. Soares, C. Albajar, J. F. de Trocóniz, M. Missiroli, D. Moran, J. Cuevas, J. Fernandez Menendez, S. Folgueras, I. Gonzalez Caballero, E. Palencia Cortezon, J. M. Vizan Garcia, I. J. Cabrillo, A. Calderon, J. R. Castiñeiras De Saa, P. De Castro Manzano, M. Fernandez, J. Garcia-Ferrero, G. Gomez, A. Lopez Virto, J. Marco, R. Marco, C. Martinez Rivero, F. Matorras, J. Piedra Gomez, T. Rodrigo, A. Y. Rodríguez-Marrero, A. Ruiz-Jimeno, L. Scodellaro, N. Trevisani, I. Vila, R. Vilar Cortabitarte, D. Abbaneo, E. Auffray, G. Auzinger, M. Bachtis, P. Baillon, A. H. Ball, D. Barney, A. Benaglia, J. Bendavid, L. Benhabib, J. F. Benitez, G. M. Berruti, P. Bloch, A. Bocci, A. Bonato, C. Botta, H. Breuker, T. Camporesi, R. Castello, G. Cerminara, M. D’Alfonso, D. d’Enterria, A. Dabrowski, V. Daponte, A. David, M. De Gruttola, F. De Guio, A. De Roeck, S. De Visscher, E. Di Marco, M. Dobson, M. Dordevic, B. Dorney, T. du Pree, D. Duggan, M. Dünser, N. Dupont, A. Elliott-Peisert, G. Franzoni, J. Fulcher, W. Funk, D. Gigi, K. Gill, D. Giordano, M. Girone, F. Glege, R. Guida, S. Gundacker, M. Guthoff, J. Hammer, P. Harris, J. Hegeman, V. Innocente, P. Janot, H. Kirschenmann, M. J. Kortelainen, K. Kousouris, K. Krajczar, P. Lecoq, C. Lourenço, M. T. Lucchini, N. Magini, L. Malgeri, M. Mannelli, A. Martelli, L. Masetti, F. Meijers, S. Mersi, E. Meschi, F. Moortgat, S. Morovic, M. Mulders, M. V. Nemallapudi, H. Neugebauer, S. Orfanelli, L. Orsini, L. Pape, E. Perez, M. Peruzzi, A. Petrilli, G. Petrucciani, A. Pfeiffer, D. Piparo, A. Racz, T. Reis, G. Rolandi, M. Rovere, M. Ruan, H. Sakulin, C. Schäfer, C. Schwick, M. Seidel, A. Sharma, P. Silva, M. Simon, P. Sphicas, J. Steggemann, B. Stieger, M. Stoye, Y. Takahashi, D. Treille, A. Triossi, A. Tsirou, G. I. Veres, N. Wardle, H. K. Wöhri, A. Zagozdzinska, W. D. Zeuner, W. Bertl, K. Deiters, W. Erdmann, R. Horisberger, Q. Ingram, H. C. Kaestli, D. Kotlinski, U. Langenegger, D. Renker, T. Rohe, F. Bachmair, L. Bäni, L. Bianchini, B. Casal, G. Dissertori, M. Dittmar, M. Donegà, P. Eller, C. Grab, C. Heidegger, D. Hits, J. Hoss, G. Kasieczka, W. Lustermann, B. Mangano, M. Marionneau, P. Martinez Ruiz del Arbol, M. Masciovecchio, D. Meister, F. Micheli, P. Musella, F. Nessi-Tedaldi, F. Pandolfi, J. Pata, F. Pauss, L. Perrozzi, M. Quittnat, M. Rossini, A. Starodumov, M. Takahashi, V. R. Tavolaro, K. Theofilatos, R. Wallny, T. K. Aarrestad, C. Amsler, L. Caminada, M. F. Canelli, V. Chiochia, A. De Cosa, C. Galloni, A. Hinzmann, T. Hreus, B. Kilminster, C. Lange, J. Ngadiuba, D. Pinna, P. Robmann, F. J. Ronga, D. Salerno, Y. Yang, M. Cardaci, K. H. Chen, T. H. Doan, Sh. Jain, R. Khurana, M. Konyushikhin, C. M. Kuo, W. Lin, Y. J. Lu, S. S. Yu, Arun Kumar, R. Bartek, P. Chang, Y. H. Chang, Y. Chao, K. F. Chen, P. H. Chen, C. Dietz, F. Fiori, U. Grundler, W.-S. Hou, Y. Hsiung, Y. F. Liu, R.-S. Lu, M. Miñano Moya, E. Petrakou, J. f. Tsai, Y. M. Tzeng, B. Asavapibhop, K. Kovitanggoon, G. Singh, N. Srimanobhas, N. Suwonjandee, A. Adiguzel, M. N. Bakirci, S. Cerci, Z. S. Demiroglu, C. Dozen, E. Eskut, F. H. Gecit, S. Girgis, G. Gokbulut, Y. Guler, Y. Guler, E. Gurpinar, I. Hos, E. E. Kangal, G. Onengut, M. Ozcan, K. Ozdemir, A. Polatoz, D. Sunar Cerci, H. Topakli, M. Vergili, C. Zorbilmez, I. V. Akin, B. Bilin, S. Bilmis, B. Isildak, G. Karapinar, M. Yalvac, M. Zeyrek, E. Gülmez, M. Kaya, O. Kaya, E. A. Yetkin, T. Yetkin, A. Cakir, K. Cankocak, S. Sen, F. I. Vardarlı, B. Grynyov, L. Levchuk, P. Sorokin, R. Aggleton, F. Ball, L. Beck, J. J. Brooke, E. Clement, D. Cussans, H. Flacher, J. Goldstein, M. Grimes, G. P. Heath, H. F. Heath, J. Jacob, L. Kreczko, C. Lucas, Z. Meng, D. M. Newbold, S. Paramesvaran, A. Poll, T. Sakuma, S. Seif El Nasr-storey, S. Senkin, D. Smith, V. J. Smith, K. W. Bell, A. Belyaev, C. Brew, R. M. Brown, L. Calligaris, D. Cieri, D. J. A. Cockerill, J. A. Coughlan, K. Harder, S. Harper, E. Olaiya, D. Petyt, C. H. Shepherd-Themistocleous, A. Thea, I. R. Tomalin, T. Williams, S. D. Worm, M. Baber, R. Bainbridge, O. Buchmuller, A. Bundock, D. Burton, S. Casasso, M. Citron, D. Colling, L. Corpe, N. Cripps, P. Dauncey, G. Davies, A. De Wit, M. Della Negra, P. Dunne, A. Elwood, A. Elwood, W. Ferguson, D. Futyan, G. Hall, G. Iles, M. Kenzie, R. Lane, R. Lucas, L. Lyons, A.-M. Magnan, S. Malik, J. Nash, A. Nikitenko, J. Pela, M. Pesaresi, K. Petridis, D. M. Raymond, A. Richards, A. Rose, C. Seez, A. Tapper, K. Uchida, M. Vazquez Acosta, T. Virdee, S. C. Zenz, J. E. Cole, P. R. Hobson, A. Khan, P. Kyberd, D. Leggat, D. Leslie, I. D. Reid, P. Symonds, L. Teodorescu, M. Turner, A. Borzou, K. Call, J. Dittmann, K. Hatakeyama, H. Liu, N. Pastika, T. Scarborough, Z. Wu, O. Charaf, S. I. Cooper, C. Henderson, P. Rumerio, D. Arcaro, A. Avetisyan, T. Bose, C. Fantasia, D. Gastler, P. Lawson, D. Rankin, C. Richardson, J. Rohlf, J. St. John, L. Sulak, D. Zou, J. Alimena, E. Berry, S. Bhattacharya, D. Cutts, N. Dhingra, A. Ferapontov, A. Garabedian, J. Hakala, U. Heintz, E. Laird, G. Landsberg, Z. Mao, M. Narain, S. Piperov, S. Sagir, R. Syarif, R. Breedon, G. Breto, M. Calderon De La Barca Sanchez, S. Chauhan, M. Chertok, J. Conway, R. Conway, P. T. Cox, R. Erbacher, G. Funk, M. Gardner, W. Ko, R. Lander, M. Mulhearn, D. Pellett, J. Pilot, F. Ricci-Tam, S. Shalhout, J. Smith, M. Squires, D. Stolp, M. Tripathi, S. Wilbur, R. Yohay, C. Bravo, R. Cousins, P. Everaerts, C. Farrell, A. Florent, J. Hauser, M. Ignatenko, D. Saltzberg, C. Schnaible, V. Valuev, M. Weber, K. Burt, R. Clare, J. Ellison, J. W. Gary, G. Hanson, J. Heilman, M. Ivova PANEVA, P. Jandir, E. Kennedy, F. Lacroix, O. R. Long, A. Luthra, M. Malberti, M. Olmedo Negrete, A. Shrinivas, H. Wei, S. Wimpenny, B. R. Yates, J. G. Branson, G. B. Cerati, S. Cittolin, R. T. D’Agnolo, M. Derdzinski, A. Holzner, R. Kelley, D. Klein, J. Letts, I. Macneill, D. Olivito, S. Padhi, M. Pieri, M. Sani, V. Sharma, S. Simon, M. Tadel, Y. Tu, A. Vartak, S. Wasserbaech, C. Welke, F. Würthwein, A. Yagil, G. Zevi Della Porta, J. Bradmiller-Feld, C. Campagnari, A. Dishaw, V. Dutta, K. Flowers, M. Franco Sevilla, P. Geffert, C. George, F. Golf, L. Gouskos, J. Gran, J. Incandela, N. Mccoll, S. D. Mullin, S. D. Mullin, J. Richman, D. Stuart, I. Suarez, C. West, J. Yoo, D. Anderson, A. Apresyan, A. Bornheim, J. Bunn, Y. Chen, J. Duarte, A. Mott, H. B. Newman, C. Pena, M. Pierini, M. Spiropulu, J. R. Vlimant, S. Xie, R. Y. Zhu, M. B. Andrews, V. Azzolini, A. Calamba, B. Carlson, T. Ferguson, M. Paulini, J. Russ, M. Sun, H. Vogel, I. Vorobiev, J. P. Cumalat, W. T. Ford, A. Gaz, F. Jensen, A. Johnson, M. Krohn, T. Mulholland, U. Nauenberg, K. Stenson, S. R. Wagner, J. Alexander, A. Chatterjee, J. Chaves, J. Chu, S. Dittmer, N. Eggert, N. Mirman, G. Nicolas Kaufman, J. R. Patterson, A. Rinkevicius, A. Ryd, L. Skinnari, L. Soffi, W. Sun, S. M. Tan, W. D. Teo, J. Thom, J. Thompson, J. Tucker, Y. Weng, P. Wittich, S. Abdullin, M. Albrow, G. Apollinari, S. Banerjee, L. A. T. Bauerdick, A. Beretvas, J. Berryhill, P. C. Bhat, G. Bolla, K. Burkett, J. N. Butler, H. W. K. Cheung, F. Chlebana, S. Cihangir, V. D. Elvira, I. Fisk, J. Freeman, E. Gottschalk, L. Gray, D. Green, S. Grünendahl, O. Gutsche, J. Hanlon, D. Hare, R. M. Harris, S. Hasegawa, J. Hirschauer, Z. Hu, B. Jayatilaka, S. Jindariani, M. Johnson, U. Joshi, A. W. Jung, B. Klima, B. Kreis, S. Lammel, J. Linacre, D. Lincoln, R. Lipton, T. Liu, R. Lopes De Sá, J. Lykken, K. Maeshima, J. M. Marraffino, V. I. Martinez Outschoorn, S. Maruyama, D. Mason, P. McBride, P. Merkel, K. Mishra, S. Mrenna, S. Nahn, C. Newman-Holmes, V. O’Dell, K. Pedro, O. Prokofyev, G. Rakness, E. Sexton-Kennedy, A. Soha, W. J. Spalding, L. Spiegel, N. Strobbe, L. Taylor, S. Tkaczyk, N. V. Tran, L. Uplegger, E. W. Vaandering, C. Vernieri, M. Verzocchi, R. Vidal, H. A. Weber, A. Whitbeck, D. Acosta, P. Avery, P. Bortignon, D. Bourilkov, A. Carnes, M. Carver, D. Curry, S. Das, R. D. Field, I. K. Furic, S. V. Gleyzer, J. Hugon, J. Konigsberg, A. Korytov, K. Kotov, J. F. Low, P. Ma, K. Matchev, H. Mei, P. Milenovic, G. Mitselmakher, D. Rank, R. Rossin, L. Shchutska, M. Snowball, D. Sperka, N. Terentyev, L. Thomas, J. Wang, S. Wang, J. Yelton, S. Hewamanage, S. Linn, P. Markowitz, G. Martinez, J. L. Rodriguez, J. R. Adams, A. Ackert, T. Adams, A. Askew, S. Bein, J. Bochenek, B. Diamond, J. Haas, S. Hagopian, V. Hagopian, K. F. Johnson, A. Khatiwada, H. Prosper, M. Weinberg, M. M. Baarmand, V. Bhopatkar, S. Colafranceschi, M. Hohlmann, H. Kalakhety, D. Noonan, T. Roy, F. Yumiceva, M. R. Adams, L. Apanasevich, D. Berry, R. R. Betts, I. Bucinskaite, R. Cavanaugh, O. Evdokimov, L. Gauthier, C. E. Gerber, D. J. Hofman, P. Kurt, C. O’Brien, l. D. Sandoval Gonzalez, C. Silkworth, P. Turner, N. Varelas, Z. Wu, M. Zakaria, B. Bilki, W. Clarida, K. Dilsiz, S. Durgut, R. P. Gandrajula, M. Haytmyradov, V. Khristenko, J.-P. Merlo, H. Mermerkaya, A. Mestvirishvili, A. Moeller, J. Nachtman, H. Ogul, Y. Onel, F. Ozok, A. Penzo, C. Snyder, E. Tiras, J. Wetzel, K. Yi, I. Anderson, I. Anderson, B. A. Barnett, B. Blumenfeld, N. Eminizer, D. Fehling, L. Feng, A. V. Gritsan, P. Maksimovic, C. Martin, M. Osherson, J. Roskes, A. Sady, U. Sarica, M. Swartz, M. Xiao, Y. Xin, C. You, M. Xiao, P. Baringer, A. Bean, G. Benelli, C. Bruner, R. P. Kenny, D. Majumder, D. Majumder, M. Malek, M. Murray, S. Sanders, R. Stringer, Q. Wang, A. Ivanov, K. Kaadze, S. Khalil, M. Makouski, Y. Maravin, A. Mohammadi, L. K. Saini, N. Skhirtladze, S. Toda, D. Lange, F. Rebassoo, D. Wright, C. Anelli, A. Baden, O. Baron, A. Belloni, B. Calvert, S. C. Eno, C. Ferraioli, J. A. Gomez, N. J. Hadley, S. Jabeen, S. Jabeen, R. G. Kellogg, T. Kolberg, J. Kunkle, Y. Lu, A. C. Mignerey, Y. H. Shin, A. Skuja, M. B. Tonjes, S. C. Tonwar, A. Apyan, R. Barbieri, A. Baty, K. Bierwagen, S. Brandt, K. Bierwagen, W. Busza, I. A. Cali, Z. Demiragli, L. Di Matteo, G. Gomez Ceballos, M. Goncharov, D. Gulhan, Y. Iiyama, G. M. Innocenti, M. Klute, D. Kovalskyi, Y. S. Lai, Y.-J. Lee, A. Levin, P. D. Luckey, A. C. Marini, C. Mcginn, C. Mironov, S. Narayanan, X. Niu, C. Paus, D. Ralph, C. Roland, G. Roland, J. Salfeld-Nebgen, G. S. F. Stephans, K. Sumorok, M. Varma, D. Velicanu, J. Veverka, J. Wang, T. W. Wang, B. Wyslouch, M. Yang, V. Zhukova, B. Dahmes, A. Evans, A. Finkel, A. Gude, P. Hansen, S. Kalafut, S. C. Kao, K. Klapoetke, Y. Kubota, Z. Lesko, J. Mans, S. Nourbakhsh, N. Ruckstuhl, R. Rusack, N. Tambe, J. Turkewitz, J. G. Acosta, S. Oliveros, E. Avdeeva, K. Bloom, S. Bose, D. R. Claes, A. Dominguez, C. Fangmeier, R. Gonzalez Suarez, R. Kamalieddin, J. Keller, D. Knowlton, I. Kravchenko, F. Meier, J. Monroy, F. Ratnikov, J. E. Siado, G. R. Snow, M. Alyari, J. Dolen, J. George, A. Godshalk, C. Harrington, I. Iashvili, J. Kaisen, A. Kharchilava, A. Kumar, S. Rappoccio, B. Roozbahani, G. Alverson, E. Barberis, D. Baumgartel, M. Chasco, A. Hortiangtham, A. Massironi, D. M. Morse, D. Nash, T. Orimoto, R. Teixeira De Lima, D. Trocino, R.-J. Wang, D. Wood, J. Zhang, K. A. Hahn, A. Kubik, N. Mucia, N. Odell, B. Pollack, A. Pozdnyakov, M. Schmitt, S. Stoynev, K. Sung, M. Trovato, M. Velasco, A. Brinkerhoff, N. Dev, M. Hildreth, C. Jessop, D. J. Karmgard, N. Kellams, K. Lannon, N. Marinelli, F. Meng, C. Mueller, Y. Musienko, M. Planer, A. Reinsvold, R. Ruchti, G. Smith, S. Taroni, N. Valls, M. Wayne, M. Wolf, A. Woodard, L. Antonelli, J. Brinson, B. Bylsma, L. S. Durkin, S. Flowers, A. Hart, C. Hill, R. Hughes, W. Ji, T. Y. Ling, B. Liu, W. Luo, D. Puigh, M. Rodenburg, B. L. Winer, H. W. Wulsin, O. Driga, P. Elmer, J. Hardenbrook, P. Hebda, S. A. Koay, P. Lujan, D. Marlow, T. Medvedeva, M. Mooney, J. Olsen, C. Palmer, P. Piroué, H. Saka, D. Stickland, C. Tully, A. Zuranski, S. Malik, V. E. Barnes, D. Benedetti, D. Bortoletto, L. Gutay, M. K. Jha, M. Jones, K. Jung, D. H. Miller, N. Neumeister, F. Primavera, B. C. Radburn-Smith, X. Shi, I. Shipsey, D. Silvers, J. Sun, A. Svyatkovskiy, F. Wang, W. Xie, L. Xu, N. Parashar, J. Stupak, A. Adair, B. Akgun, Z. Chen, K. M. Ecklund, F. J. M. Geurts, M. Guilbaud, W. Li, B. Michlin, M. Northup, B. P. Padley, R. Redjimi, J. Roberts, J. Rorie, Z. Tu, J. Zabel, B. Betchart, A. Bodek, P. de Barbaro, R. Demina, Y. Eshaq, T. Ferbel, M. Galanti, M. Galanti, A. Garcia-Bellido, J. Han, A. Harel, O. Hindrichs, O. Hindrichs, A. Khukhunaishvili, G. Petrillo, P. Tan, M. Verzetti, S. Arora, A. Barker, J. P. Chou, C. Contreras-Campana, E. Contreras-Campana, D. Ferencek, Y. Gershtein, R. Gray, E. Halkiadakis, D. Hidas, E. Hughes, S. Kaplan, R. Kunnawalkam Elayavalli, A. Lath, K. Nash, S. Panwalkar, M. Park, S. Salur, S. Schnetzer, D. Sheffield, S. Somalwar, R. Stone, S. Thomas, P. Thomassen, M. Walker, M. Foerster, G. Riley, K. Rose, S. Spanier, A. York, O. Bouhali, A. Castaneda Hernandez, A. Celik, M. Dalchenko, M. De Mattia, A. Delgado, S. Dildick, S. Dildick, R. Eusebi, J. Gilmore, T. Huang, T. Kamon, V. Krutelyov, V. Krutelyov, R. Mueller, I. Osipenkov, Y. Pakhotin, R. Patel, R. Patel, A. Perloff, A. Rose, A. Safonov, A. Tatarinov, K. A. Ulmer, N. Akchurin, C. Cowden, J. Damgov, C. Dragoiu, P. R. Dudero, J. Faulkner, S. Kunori, K. Lamichhane, S. W. Lee, T. Libeiro, S. Undleeb, I. Volobouev, E. Appelt, A. G. Delannoy, S. Greene, A. Gurrola, R. Janjam, W. Johns, C. Maguire, Y. Mao, A. Melo, H. Ni, P. Sheldon, B. Snook, S. Tuo, J. Velkovska, Q. Xu, M. W. Arenton, B. Cox, B. Francis, J. Goodell, R. Hirosky, A. Ledovskoy, H. Li, C. Lin, C. Neu, T. Sinthuprasith, X. Sun, Y. Wang, E. Wolfe, J. Wood, F. Xia, C. Clarke, R. Harr, P. E. Karchin, C. Kottachchi Kankanamge Don, P. Lamichhane, J. Sturdy, D. A. Belknap, D. Carlsmith, M. Cepeda, S. Dasu, L. Dodd, S. Duric, B. Gomber, M. Grothe, R. Hall-Wilton, M. Herndon, A. Hervé, P. Klabbers, A. Lanaro, A. Levine, K. Long, R. Loveless, A. Mohapatra, I. Ojalvo, T. Perry, G. A. Pierro, G. Polese, T. Ruggles, T. Sarangi, A. Savin, A. Sharma, N. Smith, W. H. Smith, D. Taylor, N. Woods, CMS Collaboration

**Affiliations:** Yerevan Physics Institute, Yerevan, Armenia; Institut für Hochenergiephysik der OeAW, Vienna, Austria; National Centre for Particle and High Energy Physics, Minsk, Belarus; Universiteit Antwerpen, Antwerp, Belgium; Vrije Universiteit Brussel, Brussels, Belgium; Université Libre de Bruxelles, Brussels, Belgium; Ghent University, Ghent, Belgium; Université Catholique de Louvain, Louvain-la-Neuve, Belgium; Université de Mons, Mons, Belgium; Centro Brasileiro de Pesquisas Fisicas, Rio de Janeiro, Brazil; Universidade do Estado do Rio de Janeiro, Rio de Janeiro, Brazil; Universidade Estadual Paulista, Universidade Federal do ABC, São Paulo, Brazil; Institute for Nuclear Research and Nuclear Energy, Sofia, Bulgaria; University of Sofia, Sofia, Bulgaria; Institute of High Energy Physics, Beijing, China; State Key Laboratory of Nuclear Physics and Technology, Peking University, Beijing, China; Universidad de Los Andes, Bogota, Colombia; Faculty of Electrical Engineering, Mechanical Engineering and Naval Architecture, University of Split, Split, Croatia; Faculty of Science, University of Split, Split, Croatia; Institute Rudjer Boskovic, Zagreb, Croatia; University of Cyprus, Nicosia, Cyprus; Charles University, Prague, Czech Republic; Academy of Scientific Research and Technology of the Arab Republic of Egypt, Egyptian Network of High Energy Physics, Cairo, Egypt; National Institute of Chemical Physics and Biophysics, Tallinn, Estonia; Department of Physics, University of Helsinki, Helsinki, Finland; Helsinki Institute of Physics, Helsinki, Finland; Lappeenranta University of Technology, Lappeenranta, Finland; DSM/IRFU, CEA/Saclay, Gif-sur-Yvette, France; Laboratoire Leprince-Ringuet, Ecole Polytechnique, IN2P3-CNRS, Palaiseau, France; Institut Pluridisciplinaire Hubert Curien, Université de Strasbourg, Université de Haute Alsace Mulhouse, CNRS/IN2P3, Strasbourg, France; Centre de Calcul de l’Institut National de Physique Nucleaire et de Physique des Particules, CNRS/IN2P3, Villeurbanne, France; Institut de Physique Nucléaire de Lyon, Université de Lyon, Université Claude Bernard Lyon 1, CNRS-IN2P3, Villeurbanne, France; Georgian Technical University, Tbilisi, Georgia; Tbilisi State University, Tbilisi, Georgia; I. Physikalisches Institut, RWTH Aachen University, Aachen, Germany; III. Physikalisches Institut A, RWTH Aachen University, Aachen, Germany; III. Physikalisches Institut B, RWTH Aachen University, Aachen, Germany; Deutsches Elektronen-Synchrotron, Hamburg, Germany; University of Hamburg, Hamburg, Germany; Institut für Experimentelle Kernphysik, Karlsruhe, Germany; Institute of Nuclear and Particle Physics (INPP), NCSR Demokritos, Aghia Paraskevi, Greece; University of Athens, Athens, Greece; University of Ioánnina, Ioánnina, Greece; Wigner Research Centre for Physics, Budapest, Hungary; Institute of Nuclear Research ATOMKI, Debrecen, Hungary; University of Debrecen, Debrecen, Hungary; National Institute of Science Education and Research, Bhubaneswar, India; Panjab University, Chandigarh, India; University of Delhi, Delhi, India; Saha Institute of Nuclear Physics, Kolkata, India; Bhabha Atomic Research Centre, Mumbai, India; Tata Institute of Fundamental Research, Mumbai, India; Indian Institute of Science Education and Research (IISER), Pune, India; Institute for Research in Fundamental Sciences (IPM), Tehran, Iran; University College Dublin, Dublin, Ireland; INFN Sezione di Bari, Università di Bari, Politecnico di Bari, Bari, Italy; INFN Sezione di Bologna, Università di Bologna, Bologna, Italy; INFN Sezione di Catania, Università di Catania, Catania, Italy; INFN Sezione di Firenze, Università di Firenze, Florence, Italy; INFN Laboratori Nazionali di Frascati, Frascati, Italy; INFN Sezione di Genova, Università di Genova, Genoa, Italy; INFN Sezione di Milano-Bicocca, Università di Milano-Bicocca, Milan, Italy; INFN Sezione di Napoli, Università di Napoli ‘Federico II’, Napoli, Italy, Università della Basilicata, Potenza, Italy, Università G. Marconi, Rome, Italy; INFN Sezione di Padova, Università di Padova, Padova, Italy, Università di Trento, Trento, Italy; INFN Sezione di Pavia, Università di Pavia, Pavia, Italy; INFN Sezione di Perugia, Università di Perugia, Perugia, Italy; INFN Sezione di Pisa, Università di Pisa, Scuola Normale Superiore di Pisa, Pisa, Italy; INFN Sezione di Roma, Università di Roma, Rome, Italy; INFN Sezione di Torino, Università di Torino, Torino, Italy, Università del Piemonte Orientale, Novara, Italy; INFN Sezione di Trieste, Università di Trieste, Trieste, Italy; Kangwon National University, Chunchon, Korea; Kyungpook National University, Daegu, Korea; Chonbuk National University, Jeonju, Korea; Chonnam National University, Institute for Universe and Elementary Particles, Kwangju, Korea; Korea University, Seoul, Korea; Seoul National University, Seoul, Korea; University of Seoul, Seoul, Korea; Sungkyunkwan University, Suwon, Korea; Vilnius University, Vilnius, Lithuania; National Centre for Particle Physics, Universiti Malaya, Kuala Lumpur, Malaysia; Centro de Investigacion y de Estudios Avanzados del IPN, Mexico City, Mexico; Universidad Iberoamericana, Mexico City, Mexico; Benemerita Universidad Autonoma de Puebla, Puebla, Mexico; Universidad Autónoma de San Luis Potosí, San Luis Potosí, Mexico; University of Auckland, Auckland, New Zealand; University of Canterbury, Christchurch, New Zealand; National Centre for Physics, Quaid-I-Azam University, Islamabad, Pakistan; National Centre for Nuclear Research, Swierk, Poland; Institute of Experimental Physics, Faculty of Physics, University of Warsaw, Warsaw, Poland; Laboratório de Instrumentação e Física Experimental de Partículas, Lisbon, Portugal; Joint Institute for Nuclear Research, Dubna, Russia; Petersburg Nuclear Physics Institute, Gatchina, St. Petersburg, Russia; Institute for Nuclear Research, Moscow, Russia; Institute for Theoretical and Experimental Physics, Moscow, Russia; National Research Nuclear University ‘Moscow Engineering Physics Institute’(MEPhI), Moscow, Russia; P. N. Lebedev Physical Institute, Moscow, Russia; Skobeltsyn Institute of Nuclear Physics, Lomonosov Moscow State University, Moscow, Russia; State Research Center of Russian Federation, Institute for High Energy Physics, Protvino, Russia; Faculty of Physics and Vinca Institute of Nuclear Sciences, University of Belgrade, Belgrade, Serbia; Centro de Investigaciones Energéticas Medioambientales y Tecnológicas (CIEMAT), Madrid, Spain; Universidad Autónoma de Madrid, Madrid, Spain; Universidad de Oviedo, Oviedo, Spain; Instituto de Física de Cantabria (IFCA), CSIC-Universidad de Cantabria, Santander, Spain; CERN, European Organization for Nuclear Research, Geneva, Switzerland; Paul Scherrer Institut, Villigen, Switzerland; Institute for Particle Physics, ETH Zurich, Zurich, Switzerland; Universität Zürich, Zurich, Switzerland; National Central University, Chung-Li, Taiwan; National Taiwan University (NTU), Taipei, Taiwan; Department of Physics, Faculty of Science, Chulalongkorn University, Bangkok, Thailand; Cukurova University, Adana, Turkey; Physics Department, Middle East Technical University, Ankara, Turkey; Bogazici University, Istanbul, Turkey; Istanbul Technical University, Istanbul, Turkey; Institute for Scintillation Materials of National Academy of Science of Ukraine, Kharkov, Ukraine; National Scientific Center, Kharkov Institute of Physics and Technology, Kharkov, Ukraine; University of Bristol, Bristol, UK; Rutherford Appleton Laboratory, Didcot, UK; Imperial College, London, UK; Brunel University, Uxbridge, UK; Baylor University, Waco, USA; The University of Alabama, Tuscaloosa, USA; Boston University, Boston, USA; Brown University, Providence, USA; University of California, Davis, Davis, USA; University of California, Los Angeles, USA; University of California, Riverside, Riverside, USA; University of California, San Diego, La Jolla, USA; University of California, Santa Barbara, Santa Barbara, USA; California Institute of Technology, Pasadena, USA; Carnegie Mellon University, Pittsburgh, USA; University of Colorado Boulder, Boulder, USA; Cornell University, Ithaca, USA; Fermi National Accelerator Laboratory, Batavia, USA; University of Florida, Gainesville, USA; Florida International University, Miami, USA; Florida State University, Tallahassee, USA; Florida Institute of Technology, Melbourne, USA; University of Illinois at Chicago (UIC), Chicago, USA; The University of Iowa, Iowa City, USA; Johns Hopkins University, Baltimore, USA; The University of Kansas, Lawrence, USA; Kansas State University, Manhattan, USA; Lawrence Livermore National Laboratory, Livermore, USA; University of Maryland, College Park, USA; Massachusetts Institute of Technology, Cambridge, USA; University of Minnesota, Minneapolis, USA; University of Mississippi, Oxford, USA; University of Nebraska-Lincoln, Lincoln, USA; State University of New York at Buffalo, Buffalo, USA; Northeastern University, Boston, USA; Northwestern University, Evanston, USA; University of Notre Dame, Notre Dame, USA; The Ohio State University, Columbus, USA; Princeton University, Princeton, USA; University of Puerto Rico, Mayagüez, USA; Purdue University, West Lafayette, USA; Purdue University Calumet, Hammond, USA; Rice University, Houston, USA; University of Rochester, Rochester, USA; Rutgers, The State University of New Jersey, Piscataway, USA; University of Tennessee, Knoxville, USA; Texas A&M University, College Station, USA; Texas Tech University, Lubbock, USA; Vanderbilt University, Nashville, USA; University of Virginia, Charlottesville, USA; Wayne State University, Detroit, USA; University of Wisconsin, Madison, USA; CERN, Geneva, Switzerland

## Abstract

New sets of parameters (“tunes”) for the underlying-event (UE) modelling of the pythia8, pythia6 and herwig++ Monte Carlo event generators are constructed using different parton distribution functions. Combined fits to CMS UE proton–proton ($\mathrm {p}\mathrm {p}$) data at $\sqrt{s} = 7\,\text {TeV}$ and to UE proton–antiproton ($\mathrm {p}\overline{\mathrm{p}}$) data from the CDF experiment at lower $\sqrt{s}$, are used to study the UE models and constrain their parameters, providing thereby improved predictions for proton–proton collisions at 13$\,\text {TeV}$. In addition, it is investigated whether the values of the parameters obtained from fits to UE observables are consistent with the values determined from fitting observables sensitive to double-parton scattering processes. Finally, comparisons are presented of the UE tunes to “minimum bias” (MB) events, multijet, and Drell–Yan ($\mathrm{q} \overline{\mathrm{q}} \rightarrow \mathrm{Z}/ \gamma ^* \rightarrow$ lepton-antilepton+jets) observables at 7 and 8$\,\text {TeV}$, as well as predictions for MB and UE observables at 13$\,\text {TeV}$.

## Introduction

Monte Carlo (MC) event generators of hadron–hadron collisions based on perturbative quantum chromodynamics (QCD) contain several components. The “hard-scattering” part of the event consists of particles resulting from the hadronization of the two partons (jets) produced in the hardest scattering, and in their associated hard initial- and final-state radiation (ISR and FSR). The underlying event (UE) consists of particles from the hadronization of beam-beam remnants (BBR), of multiple-parton interactions (MPI), and their associated ISR and FSR. The BBR include hadrons from the fragmentation of spectator partons that do not exchange any appreciable transverse momentum ($p_{\mathrm {T}}$) in the collision. The MPI are additional 2-to-2 parton-parton scatterings that occur within the same hadron–hadron collision, and are softer in transverse momentum ($p_{\mathrm {T}} \lesssim 3\,\text {GeV}$) than the hard scattering.

The perturbative 2-to-2 parton-parton differential cross section diverges like $1/\hat{p}_\mathrm{T}^4$, where $\hat{p}_\mathrm{T}$ is the transverse momentum of the outgoing partons in the parton-parton center-of-mass (c.m.) frame. Usually, QCD MC models such as pythia  [1–5] regulate this divergence by including a smooth phenomenological cutoff $p_\mathrm{T0}$ as follows:

1 $$\begin{aligned} 1/\hat{p}_\mathrm{T}^4\rightarrow 1/(\hat{p}_\mathrm{T}^2+p_\mathrm{T0}^2)^2. \end{aligned}$$

This formula approaches the perturbative result for large scales and is finite as $\hat{p}_\mathrm{T}\rightarrow 0$. The divergence of the strong coupling $\alpha _\mathrm{s}$ at low $\hat{p}_\mathrm{T}$ is also regulated through Eq. (1). The primary hard 2-to-2 parton-parton scattering process and the MPI are regulated in the same way through a single $p_\mathrm{T0}$ parameter. However, this cutoff is expected to have a dependence on the center-of-mass energy of the hadron–hadron collision $\sqrt{s}$. In the pythia MC event generator this energy dependence is parametrized with a power-law function with exponent $\epsilon$:

2 $$\begin{aligned} p_\mathrm{T0}(\sqrt{s})= p_\mathrm{T0}^\mathrm{ref} \, (\sqrt{s}/\sqrt{s_0})^\epsilon , \end{aligned}$$

where $\sqrt{s_0}$ is a given reference energy and $p_\mathrm{T0}^\mathrm{ref}$ is the value of $p_\mathrm{T0}$ at $\sqrt{s_0}$. At a given $\sqrt{s}$, the amount of MPI depends on $p_\mathrm{T0}$, the parton distribution functions (PDF), and the overlap of the matter distributions (or centrality) of the two colliding hadrons. Smaller values of $p_\mathrm{T0}$ provide more MPI due to a larger MPI cross section. Table 1 shows the parameters in pythia6 [1] and pythia8 [5] that, together with the selected PDF, determine the energy dependence of MPI. Recently, in herwig++ [6, 7] the same formula has been adopted to provide an energy dependence to their MPI cutoff, which is also shown in Table 1. The QCD MC generators have other parameters that can be adjusted to control the modelling of the properties of the events, and a specified set of such parameters adjusted to fit certain prescribed aspects of the data is referred to as a “tune” [8–10].

**Table 1:** Parameters in pythia6 [1], pythia8 [5], and herwig++ [6, 7] MC event generators that, together with some chosen PDF, determine the energy dependence of MPI

Parameter	pythia6	pythia8	herwig++
MPI cutoff, $p_\mathrm{T0}^\mathrm{ref}$, at $\sqrt{s} = \sqrt{s_0}$	PARP(82)	MultipartonInteractions:pT0Ref	MPIHandler:pTmin0
Reference energy, $\sqrt{s_0}$	PARP(89)	MultipartonInteractions:ecmRef	MPIHandler:ReferenceScale
Exponent of $\sqrt{s}$ dependence, $\epsilon$	PARP(90)	MultipartonInteractions:ecmPow	MPIHandler:Power

In addition to hard-scattering processes, other processes contribute to the inelastic cross section in hadron–hadron collisions: single-diffraction dissociation (SD), double-diffraction dissociation (DD), and central-diffraction (CD). In SD and DD events, one or both beam particles are excited into high-mass color-singlet states (i.e.  into some resonant ${\mathrm {N}}^*$), which then decay. The SD and DD processes correspond to color-singlet exchanges between the beam hadrons, while CD corresponds to double color-singlet exchange with a diffractive system produced centrally. For non-diffractive processes (ND), color is exchanged, the outgoing remnants are no longer color singlets, and this separation of color generates a multitude of quark–antiquark pairs that are created via vacuum polarization. The sum of all components except SD corresponds to non single-diffraction (NSD) processes.

Minimum bias (MB) is a generic term that refers to events selected by requiring minimal activity within the detector. This selection accepts a large fraction of the overall inelastic cross section. Studies of the UE are often based on MB data, but it should be noted that the dominant particle production mechanisms in MB collisions and in the UE are not exactly the same. On the one hand, the UE is studied in collisions in which a hard 2-to-2 parton-parton scattering has occurred, by analyzing the hadronic activity in different regions of the event relative to the back-to-back azimuthal structure of the hardest particles emitted [11]. On the other hand, MB collisions are often softer and include diffractive interactions that, in the case of pythia, are modelled via a Regge-based approach [12].

The MPI are usually much softer than primary hard scatters, however, occasionally two hard 2-to-2 parton scatters can take place within the same hadron–hadron collision. This is referred to as double-parton scattering (DPS) [13–16], and is typically described in terms of an effective cross section parameter, $\sigma _\mathrm{eff}$, defined as:

3 $$\begin{aligned} \sigma _\mathrm{AB} = \frac{\sigma _\mathrm{A} \sigma _\mathrm{B}}{\sigma _\mathrm{eff}}, \end{aligned}$$

where $\sigma _\mathrm{A}$ and $\sigma _\mathrm{B}$ are the inclusive cross sections for individual hard scattering processes of generic type A and B, respectively, and $\sigma _\mathrm{AB}$ is the cross section for producing both scatters in the same hadron–hadron collision. If A and B are indistinguishable, as in four-jet production, a statistical factor of 1 / 2 must be inserted on the right-hand side of Eq. (3). Furthermore, $\sigma _\mathrm{eff}$ is assumed to be independent of A and B. However, $\sigma _\mathrm{eff}$ is not a directly observed quantity, but can be calculated from the overlap function of the two transverse profile distributions of the colliding hadrons, as implemented in any given MPI model.

The UE tunes have impact in both soft and hard particle production in a given pp collision. First, about half of the particles produced in a MB collision originate from the hadronization of partons scattered in MPI, and have their differential cross sections in $p_{\mathrm {T}}$ regulated via Eq. (1), using the same $p_\mathrm{T0}$ cutoff used to tame the hardest 2-to-2 parton-parton scattering in the event. The tuning of the cross-section regularization affects therefore all (soft and hard) parton-parton scatterings and provides a prediction for the behavior of the ND cross section. Second, the UE tunes parametrize the distribution in the transverse overlap of the colliding protons and thereby the probability of two hard parton-parton scatters that is then used to estimate DPS-sensitive observables.

In this paper, we study the $\sqrt{s}$ dependence of the UE using recent CDF proton–antiproton data from the Fermilab Tevatron at 0.3, 0.9, and $1.96\,\text {TeV}$ [11], together with CMS pp data from the CERN LHC at $\sqrt{s} = 7\,\text {TeV}$ [17]. The 0.3 and $0.9\,\text {TeV}$ data are from the “Tevatron energy scan” performed just before the Tevatron was shut down. Using the rivet (version 1.9.0) and professor (version 1.3.3) frameworks [18, 19], we construct: (i) new pythia8 (version 8.185) UE tunes using several PDF sets (CTEQ6L1 [20], HERAPDF$1.5$LO [21], and NNPDF2.3LO [22, 23]), (ii) new pythia6 (version 6.327) UE tunes (using CTEQ6L1 and HERAPDF$1.5$LO), and (iii) a new herwig++ (version 2.7.0) UE tune for CTEQ6L1. The rivet software is a tool for producing predictions of physics quantities obtained from MC event generators. It is used for generating sets of MC predictions with a different choice of parameters related to the UE simulation. The predictions are then included in the professor framework, which parametrizes the generator response and returns the set of tuned parameters that best fits the input measurements.

In addition, we construct several new CMS “DPS tunes” and investigate whether the values of the UE parameters determined from fitting the UE observables in a hard-scattering process are consistent with the values determined from fitting DPS-sensitive observables. The professor software also offers the possibility of extracting “eigentunes”, which provide an estimate of the uncertainties in the fitted parameters. The eigentunes consist of a collection of additional tunes, obtained through the covariance matrix of the data-theory fitting procedure, to determine independent directions in parameter space that provide a specific modification in the goodness of the fit, $\chi ^2$ (Sect. 2). All of the CMS UE and DPS tunes are provided with eigentunes. In Sect. 4, predictions using the CMS UE tunes are compared to other UE measurements not used in determining the tunes, and we examine how well Drell–Yan, MB, and multijet observables can be predicted using the UE tunes. In Sect. 5, predictions of the new tunes are shown for UE observables at $13\,\text {TeV}$, together with a comparison to the first MB distribution measured. Section 6 has a brief summary and conclusions. The appendices contain additional comparisons between the pythia6 and herwig++ UE tunes and the data, information about the tune uncertainties, and predictions for some MB and DPS observables at 13$\,\text {TeV}$.

## The CMS UE tunes

Previous UE studies have used the charged-particle jet with largest $p_{\mathrm {T}}$ [24, 25] or a $\mathrm{Z}$ boson [11, 26] as the leading (i.e. highest $p_{\mathrm {T}}$) objects in the event. The CDF and CMS data, used for the tunes, select the charged particle with largest $p_{\mathrm {T}}$ in the event ($p_\mathrm{T}^\mathrm{max}$) as the “leading object”, and use just the charged particles with $p_\mathrm{T}\!>\!0.5\,\text {GeV}$ and $|\eta | < 0.8$ to characterize the UE.

**Fig. 1:**
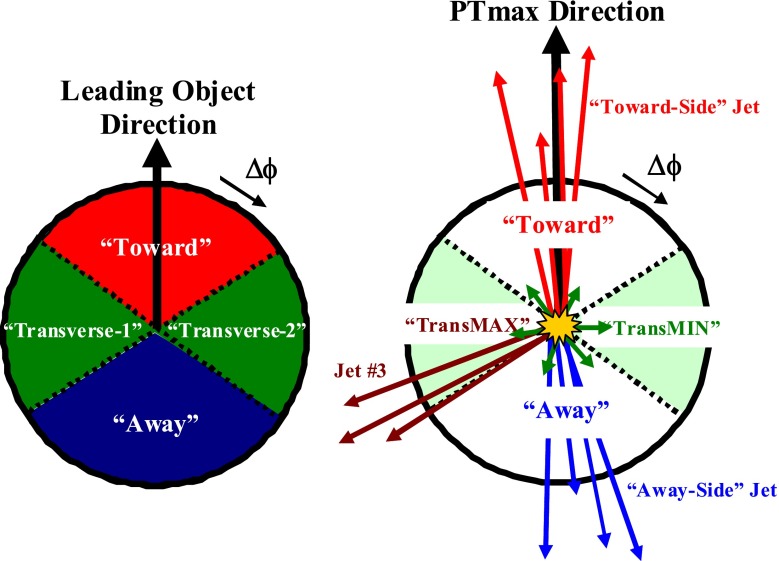
*Left* Illustration of the azimuthal regions in an event defined by the $\varDelta \phi$ angle relative to the direction of the leading object [11]. *Right* Illustration of the topology of a hadron–hadron collision in which a hard parton–parton collision has occurred, and the leading object is taken to be the charged particle of largest $p_{\mathrm {T}}$ in the event, $p_\mathrm{T}^\mathrm{max}$

On an event-by-event basis, the leading object is used to define regions of pseudorapidity-azimuth ($\eta$-$\phi$) space. The “toward” region relative to this direction, as indicated in Fig. 1, is defined by $|\varDelta \phi |<\pi /3$ and $|\eta | < 0.8$, and the “away” region by $|\varDelta \phi |>2\pi /3$ and $|\eta | < 0.8$. The charged-particle and the scalar-$p_{\mathrm {T}}$ sum densities in the transverse region are calculated as the sum of the contribution in the two regions: “Transverse-1” ($\pi /3<\varDelta \phi <2\pi /3$, $|\eta | < 0.8$) and “Transverse-2” ($\pi /3<-\varDelta \phi <2\pi /3$, $|\eta | < 0.8$), divided by the area in $\eta$-$\phi$ space, $\varDelta \eta \varDelta \phi = 1.6\times 2\pi /3$. The transverse region is further separated into the “TransMAX” and “TransMIN” regions, also shown in Fig. 1. This defines on an event-by-event basis the regions with more (TransMAX) and fewer (TransMIN) charged particles (${\mathrm {N}}_\mathrm{ch}$), or greater (TransMAX) or smaller (TransMIN) scalar-$p_{\mathrm {T}}$ sums ($p_\mathrm{T}^\mathrm{sum}$). The UE particle and $p_{\mathrm {T}}$ densities are constructed by dividing by the area in $\eta$-$\phi$ space, where the TransMAX and TransMIN regions each have an area of $\varDelta \eta \varDelta \phi = 1.6\times 2\pi /6$. The transverse density (also referred to as “TransAVE”) is the average of the TransMAX and the TransMIN densities. For events with hard initial- or final-state radiation, the TransMAX region often contains a third jet, but both the TransMAX and TransMIN regions receive contributions from the MPI and beam-beam remnant components. The TransMIN region is very sensitive to the MPI and beam-beam remnant components of the UE, while “TransDIF” (the difference between TransMAX and TransMIN densities) is very sensitive to ISR and FSR [27].

The new UE tunes are determined by fitting UE observables, and using only those parameters that are most sensitive to the UE data. Since it is not possible to tune all parameters of a MC event generator at once, the parameters that affect, for example, the parton shower, the fragmentation, and the intrinsic-parton $p_{\mathrm {T}}$ are fixed to the values given by an initially established reference tune. The initial reference tunes used for pythia8 are Tune 4C [28] and the Monash Tune [29]. For pythia6, the reference tune is Tune Z2*lep [25], and for herwig++ it is Tune UE-EE-5C [30].

### The PYTHIA8 UE tunes

Taking as the reference tune the set of parameters of pythia8 Tune $4$C [28], we construct two new UE tunes, one using CTEQ6L1 (CUETP$8$S$1$-CTEQ$6$L1) and one using HERAPDF$1.5$LO (CUETP$8$S$1$-HERAPDF1.5LO). CUET (read as “cute”) stands for “CMS UE tune”, and P8S1 stands for pythia8 “Set 1”.

The tunes are extracted by varying the four parameters in Table 2 in fits to the TransMAX and TransMIN charged-particle and $p_\mathrm{T}^\mathrm{sum}$ densities at three energies, for $\mathrm {p}\overline{\mathrm{p}}$ collisions at $\sqrt{s} = 0.9$ and 1.96, and $\mathrm {p}\mathrm {p}$ collisions at $7\,\text {TeV}$. The measurements of TransAVE and TransDIF densities are not included in the fit, since they can be constructed from TransMAX and TransMIN. The new tunes use an exponentially-falling matter-overlap function between the two colliding protons of the form exp(−*b*^expPow^), with *b* being the impact parameter of the collision. The parameters that are varied are expPow, the MPI energy-dependence parameters (Table 1) and the range, i.e. the probability, of color reconnection (CR). A small (large) value of the final-state CR parameter tends to increase (reduce) the final particle multiplicities. In pythia8, unlike in pythia6, only one parameter determines the amount of CR, which includes a $p_{\mathrm {T}}$ dependence, as defined in Ref. [5].

The generated inelastic events include ND and diffractive (DD$+$SD$+$CD) contributions, although the UE observables used to determine the tunes are sensitive to single-diffraction dissociation, central-diffraction, and double-diffraction dissociation only at very small $p_\mathrm{T}^\mathrm{max}$ values (e.g. $p_\mathrm{T}^\mathrm{max}<1.5\,\text {GeV}$). The ND component dominates for $p_\mathrm{T}^\mathrm{max}$ values greater than ${\approx } 2.0\,\text {GeV}$, since the cross section of the diffractive components rapidly decreases as a function of $\hat{p}_\mathrm{T}$. The fit is performed by minimizing the $\chi ^2$ function:

4 $$\begin{aligned} \chi ^2(p)=\sum _{i}\frac{(f^{i}(p)-R_{i})^2}{\varDelta _{i}^2}, \end{aligned}$$

where the sum runs over each bin *i* of every observable. The $f^{i}(p)$ functions correspond to the interpolated MC response for the simulated observables as a function of the parameter vector *p*, $R_i$ is the value of the measured observable in bin *i*, and $\varDelta _i$ is the total experimental uncertainty of $R_i$. We do not use the Tevatron data at $\sqrt{s}=300\,\text {GeV}$, as we are unable to obtain an acceptable $\chi ^2$ in a fit of the four parameters in Table 2. The $\chi ^2$ per degree of freedom (dof) listed in Table 2 refers to the quantity $\chi ^2(p)$ in Eq. (4), divided by the number of dof in the fit. The eigentunes (Appendix A) correspond to the tunes in which the changes in the $\chi ^2$ ($\varDelta \chi ^2$) of the fit relative to the best-fit value equals the $\chi ^2$ value obtained in the tune, i.e. $\varDelta \chi ^2$ = $\chi ^2$. For both tunes in Table 2, the fit quality is very good, with $\chi ^2$/dof values very close to 1.

The contribution from CR changes in the two new tunes; it is large for the HERAPDF1.5LO and small for the CTEQ6L1 PDF. This is a result of the shape of the parton densities at small fractional momenta *x*, which is different for the two PDF sets. While the parameter $p_\mathrm{T0}^\mathrm{ref}$ in Eq. (2) stays relatively constant between Tune $4$C and the new tunes, the energy dependence $\epsilon$ tends to increase in the new tunes, as do the matter-overlap profile functions.

**Table 2:** The pythia8 parameters, tuning range, Tune $4$C values [28], and best-fit values for CUETP$8$S$1$-CTEQ$6$L1 and CUETP$8$S$1$-HERAPDF1.5LO, obtained from fits to the TransMAX and TransMIN charged-particle and $p_\mathrm{T}^\mathrm{sum}$ densities, as defined by the leading charged-particle $p_\mathrm{T}^\mathrm{max}$at $\sqrt{s} = 0.9$, 1.96, and $7\,\text {TeV}$. The $\sqrt{s}=300\,\text {GeV}$ data are excluded from the fit

pythia8 Parameter	Tuning range	Tune $4$C	CUETP8S1	CUETP8S1
PDF	–	CTEQ6L1	CTEQ6L1	HERAPDF1.5LO
MultipartonInteractions:pT0Ref [GeV]	1.0–$3.0\phantom {0}$	2.085	2.101	2.000
MultipartonInteractions:ecmPow	0.0–$0.4\phantom {0}$	$0.19\phantom {0}$	0.211	0.250
MultipartonInteractions:expPow	0.4–10.0	$2.0\phantom {00}$	1.609	1.691
ColourReconnection:range	0.0–$9.0\phantom {0}$	$1.5\phantom {00}$	3.313	6.096
MultipartonInteractions:ecmRef [GeV]	–	1800	$1800^\mathrm{a}$	$1800^\mathrm{a}$
$\chi ^2$/dof	–	–	0.952	$1.13\phantom {00}$

$^\mathrm{a}$ Fixed at Tune $4$C value

The pythia8 Monash Tune [29] combines updated fragmentation parameters with the NNPDF2.3LO PDF.

The NNPDF2.3LO PDF has a gluon distribution at small *x* that is different compared to CTEQ6L1 and HERAPDF$1.5$LO, and this affects predictions in the forward region of hadron–hadron collisions. Tunes using the NNPDF2.3LO PDF provide a more consistent description of the UE and MB observables in both the central and forward regions, than tunes using other PDF.

A new pythia8 tune CUETP$8$M$1$ (labeled with M for Monash) is constructed using the parameters of the Monash Tune and fitting the two MPI energy-dependence parameters of Table 1 to UE data at $\sqrt{s} = 0.9$, 1.96, and $7\,\text {TeV}$. Varying the CR range and the exponential slope of the matter-overlap function freely in the minimization of the $\chi ^2$ leads to suboptimal best-fit values. The CR range is therefore fixed to the value of the Monash Tune, and the exponential slope of the matter-overlap function expPow is set to 1.6, which is similar to the value determined in CUETP8S1-CTEQ6L1. The best-fit values of the two tuned parameters are shown in Table 3. Again, we exclude the $300\,\text {GeV}$ data, since we are unable to get a good $\chi ^2$ in the fit. The parameters obtained for CUETP$8$M$1$ differ slightly from the ones of the Monash Tune. The obtained energy-dependence parameter $\epsilon$ is larger, while a very similar value is obtained for $p_\mathrm{T0}^\mathrm{ref}$.

**Table 3:** The pythia8 parameters, tuning range, Monash values [29], and best-fit values for CUETP$8$M$1$, obtained from fits to the TransMAX and TransMIN charged-particle and $p_\mathrm{T}^\mathrm{sum}$ densities, as defined by the leading charged-particle $p_\mathrm{T}^\mathrm{max}$at $\sqrt{s} = 0.9$, 1.96, and $7\,\text {TeV}$. The $\sqrt{s}=300\,\text {GeV}$ data are excluded from the fit

pythia8 Parameter	Tuning range	Monash	CUETP8M1
PDF	–	NNPDF2.3LO	NNPDF2.3LO
MultipartonInteractions:pT0Ref [GeV]	1.0–3.0	2.280	2.402
MultipartonInteractions:ecmPow	0.0–0.4	0.215	0.252
MultipartonInteractions:expPow	–	$1.85\phantom {0}$	$1.6^{\mathrm{a}}\phantom {0}$
ColourReconnection:range	–	$1.80\phantom {0}$	$1.80^{\mathrm{b}}$
MultipartonInteractions:ecmRef [GeV]	–	7000	$7000^{\mathrm{b}}$
$\chi ^2$/dof	–	–	$1.54\phantom {0}$

$^{\mathrm{a}}$ Fixed at CUETP$8$S$1$-CTEQ$6$L1 value

$^{\mathrm{b}}$ Fixed at Monash Tune value

**Fig. 2:**
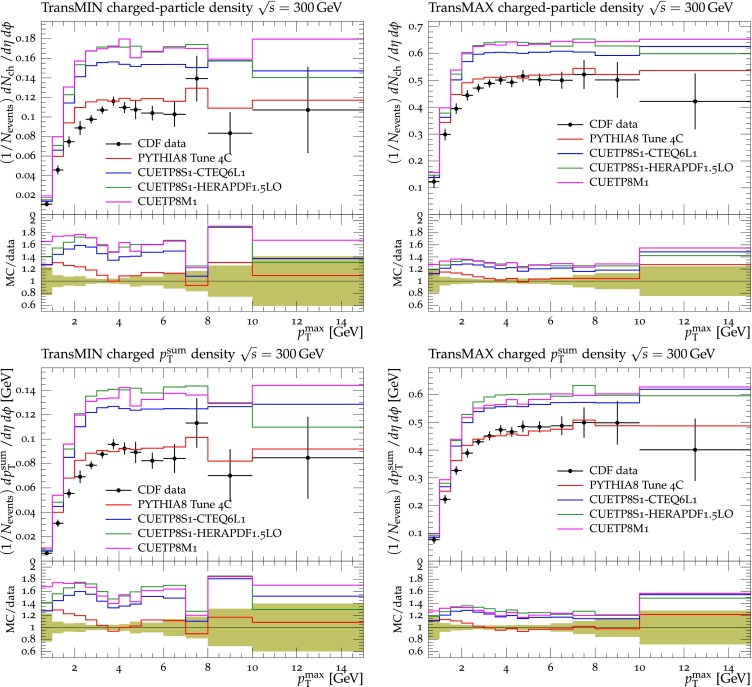
CDF data at $\sqrt{s}=300\,\text {GeV}$ [11] on particle (*top*) and $p_\mathrm{T}^\mathrm{sum}$ densities (*bottom*) for charged particles with $p_\mathrm{T}\!>\!0.5\,\text {GeV}$ and $|\eta |\!<\!0.8$ in the TransMIN (*left*) and TransMAX (*right*) regions as defined by the leading charged particle, as a function of the transverse momentum of the leading charged-particle $p_\mathrm{T}^\mathrm{max}$. The data are compared to pythia8 Tune $4$C, CUETP$8$S$1$-CTEQ$6$L1, CUETP$8$S$1$-HERAPDF1.5LO, and CUETP$8$M$1$. The ratios of MC events to data are given below each panel. The data at $\sqrt{s}=300\,\text {GeV}$ are not used in determining these tunes. The *green bands* in the ratios represent the total experimental uncertainties

**Fig. 3:**
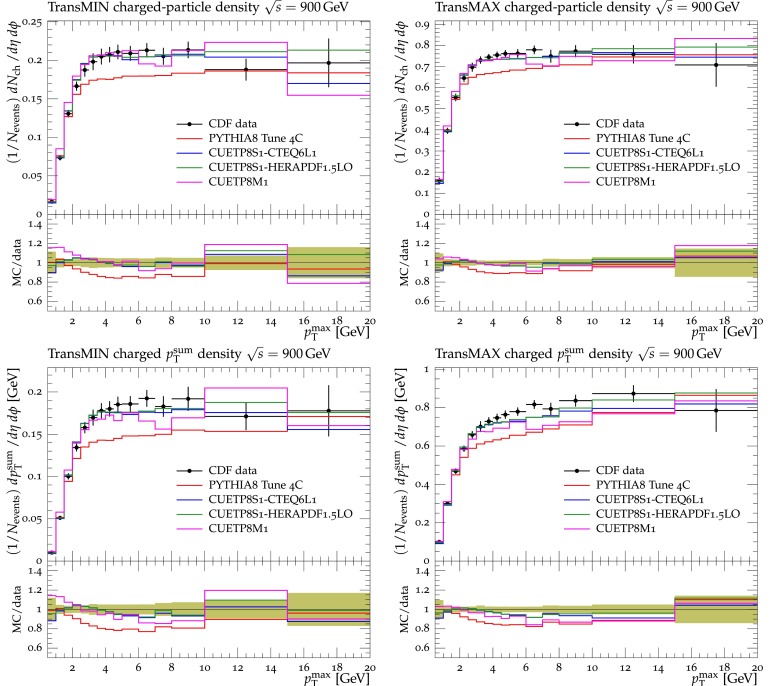
CDF data at $\sqrt{s}=900\,\text {GeV}$ [11] on particle (*top*) and $p_\mathrm{T}^\mathrm{sum}$ densities (*bottom*) for charged particles with $p_\mathrm{T}\!>\!0.5\,\text {GeV}$ and $|\eta |\!<\!0.8$ in the TransMIN (*left*) and TransMAX (*right*) regions as defined by the leading charged particle, as a function of the transverse momentum of the leading charged-particle $p_\mathrm{T}^\mathrm{max}$. The data are compared to pythia8 Tune $4$C, CUETP$8$S$1$-CTEQ$6$L1, CUETP$8$S$1$-HERAPDF1.5LO, and CUETP$8$M$1$. The ratios of MC events to data are given below each panel. The *green bands* in the ratios represent the total experimental uncertainties

**Fig. 4:**
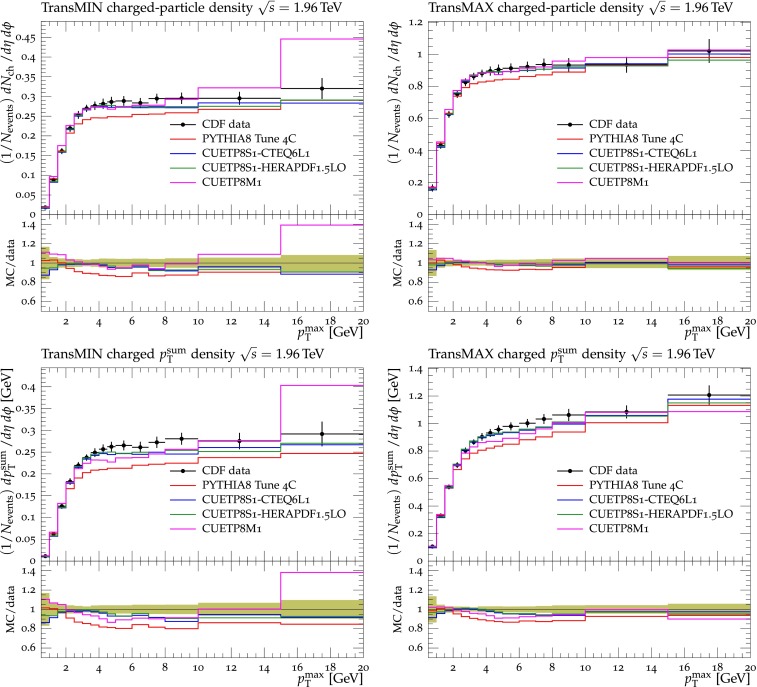
CDF data at $\sqrt{s}=1.96\,\text {TeV}$ [11] on particle (*top*) and $p_\mathrm{T}^\mathrm{sum}$ densities (*bottom*) for charged particles with $p_\mathrm{T}\!>\!0.5\,\text {GeV}$ and $|\eta |\!<\!0.8$ in the TransMIN (*left*) and TransMAX (*right*) regions as defined by the leading charged particle, as a function of the transverse momentum of the leading charged-particle $p_\mathrm{T}^\mathrm{max}$. The data are compared to pythia8 Tune $4$C, CUETP$8$S$1$-CTEQ$6$L1, CUETP$8$S$1$-HERAPDF1.5LO, and CUETP$8$M$1$. The ratios of MC events to data are given below each panel. The *green bands* in the ratios represent the total experimental uncertainties

**Fig. 5:**
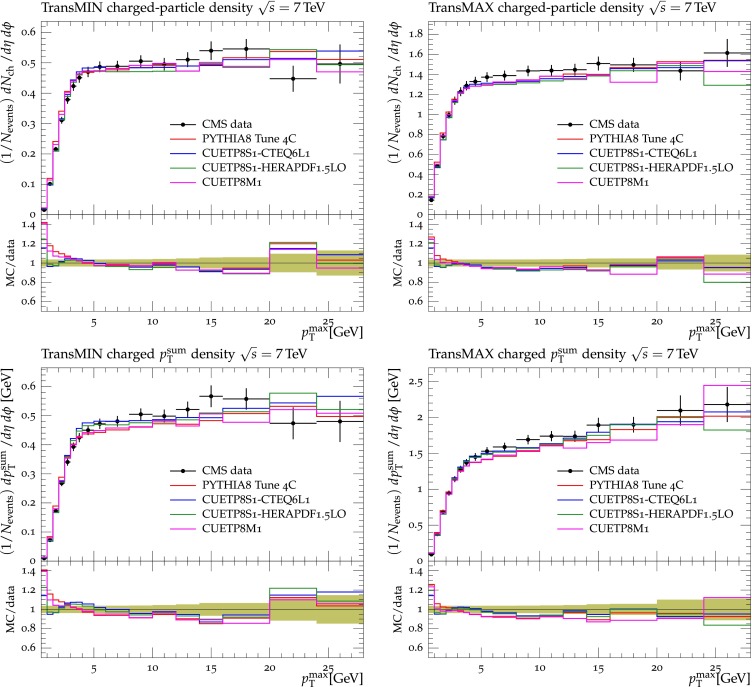
CMS data at $\sqrt{s}=7\,\text {TeV}$ [17] on particle (*top*) and $p_\mathrm{T}^\mathrm{sum}$ densities (*bottom*) for charged particles with $p_\mathrm{T}\!>\!0.5\,\text {GeV}$ and $|\eta |\!<\!0.8$ in the TransMIN (*left*) and TransMAX (*right*) regions as defined by the leading charged particle, as a function of the transverse momentum of the leading charged-particle $p_\mathrm{T}^\mathrm{max}$. The data are compared to pythia8 Tune $4$C, and CUETP$8$S$1$-CTEQ$6$L1, CUETP$8$S$1$-HERAPDF1.5LO, and CUETP$8$M$1$. The ratios of MC events to data are given below each panel. The *green bands* in the ratios represent the total experimental uncertainties

Figures 2, 3, 4 and 5 show the CDF data at 0.3, 0.9, and $1.96\,\text {TeV}$, and the CMS data at $7\,\text {TeV}$ for charged-particle and $p_\mathrm{T}^\mathrm{sum}$ densities in the TransMIN and TransMAX regions as a function of $p_\mathrm{T}^\mathrm{max}$, compared to predictions obtained with the pythia8 Tune $4$C and with the new CMS tunes: CUETP$8$S$1$-CTEQ$6$L1, CUETP$8$S$1$-HERAPDF1.5LO, and CUETP$8$M$1$. Predictions from the new tunes cannot reproduce the $\sqrt{s}=300\,\text {GeV}$ data, but describe very well the data at the higher $\sqrt{s} = 0.9$, 1.96, and $7\,\text {TeV}$. In particular, the description provided by the new tunes significantly improves relative to the old Tune $4$C, which is likely due to the better choice of parameters used in the MPI energy dependence and the extraction of the CR in the retuning.

### The PYTHIA6 UE tunes

The pythia6 Tune Z$2^*$lep [25] uses the improved fragmentation parameters from fits to the LEP e$^+$e$^-$ data [31], and a double-Gaussian matter profile for the colliding protons but corresponds to an outdated CMS UE tune. It was constructed by fitting the CMS charged-particle jet UE data at 0.9 and $7\,\text {TeV}$ [24] using data on the TransAVE charged-particle and $p_\mathrm{T}^\mathrm{sum}$ densities, since data on TransMAX, TransMIN, and TransDIF were not available at that time.

Starting with Tune Z$2^*$lep parameters, two new pythia6 UE tunes are constructed, one using CTEQ6L1 (CUETP$6$S$1$-CTEQ$6$L1) and one using HERAPDF$1.5$LO (CUETP$6$S$1$-HERAPDF1.5LO), with P6S1 standing for pythia6 “Set 1”. The tunes are constructed by fitting the five parameters shown in Table 4 to the TransMAX and TransMIN charged-particle and $p_\mathrm{T}^\mathrm{sum}$ densities at $\sqrt{s} = 0.3$, 0.9, 1.96, and $7\,\text {TeV}$. In addition to varying the MPI energy-dependence parameters (Table 1), we also vary the core-matter fraction PARP(83), which parametrizes the amount of matter contained within the radius of the proton core, the CR strength PARP(78), and the CR suppression PARP(77). The PARP(78) parameter reflects the probability for a given string to retain its color history, and therefore does not change the color and other string pieces, while the PARP(77) parameter introduces a $p_{\mathrm {T}}$ dependence on the CR probability [1].

Inelastic events (ND$+$DD$+$SD$+$CD) are generated with pythia6. The best-fit values of the five parameters are shown in Table 4. The matter-core fraction is quite different in the two new pythia6 tunes. This is due to the fact that this parameter is very sensitive to the behaviour of the PDF at small *x*. Predictions obtained with pythia6 Tune Z$2^*$lep , CUETP6S1-CTEQ6L1 and CUETP6S1-HERAPDF1.5LO are compared in Appendix B to the UE data. The new pythia6 tunes significantly improve the description of the UE data relative to pythia6 Tune Z$2^*$lep at all considered energies, due to the better choice of parameters governing the MPI energy dependence.

**Table 4:** The pythia6 parameters, tuning range, Tune Z$2^*$lep values [31], and best-fit values for CUETP$6$S$1$-CTEQ$6$L1 and CUETP$6$S$1$-HERAPDF1.5LO, obtained from fits to the TransMAX and TransMIN charged-particle and $p_\mathrm{T}^\mathrm{sum}$ densities as defined by the $p_\mathrm{T}^\mathrm{max}$ of the leading charged particle at $\sqrt{s} = 0.3$ , 0.9, 1.96, and $7\,\text {TeV}$

pythia6 Parameter	Tuning Range	Tune Z$2^*$lep	CUETP6S1	CUETP6S1
PDF	–	CTEQ6L1	CTEQ6L1	HERAPDF1.5LO
PARP(82)-MPI cutoff [GeV]	1.6–2.2	1.921	1.910	1.946
PARP(90)-exponent of $\sqrt{s}$ dependence	0.18–0.28	0.227	0.248	0.250
PARP(77)-CR suppression	0.25–1.15	1.016	0.665	0.667
PARP(78)-CR strength	0.2–0.8	0.538	0.545	0.537
PARP(83)-matter fraction in core	0.1–1.0	0.356	0.822	0.490
PARP(89)-reference energy [GeV]	–	1800	$1800^\mathrm{a}$	$1800^\mathrm{a}$
$\chi ^2$/dof	–	–	0.915	1.004

$^\mathrm{a}$ Fixed at Tune Z$2^*$lep value

### The HERWIG++ UE tunes

Starting with the parameters of herwig++ Tune UE-EE-5C [30], we construct a new herwig++ UE tune, CUETHppS$$1$$, where Hpp stands for herwig++. This tune is obtained by varying the four parameters shown in Table 5 in the fit to TransMAX and TransMIN charged-particle and $$p_\mathrm{T}^\mathrm{sum}$$ densities at the four $$\sqrt{s} = 0.3$$, 0.9, 1.96, and $$7\,\text {TeV} $$. We set the MPI cutoff $$p_\mathrm{T0}$$ and the reference energy $$\sqrt{s_0}$$ to the Tune UE-EE-5C values, and vary the MPI c.m. energy extrapolation parameter in Table 1. We also vary the inverse radius that determines the matter overlap and the range of CR. The CR model in herwig++ is defined by two parameters, one (colourDisrupt) ruling the color structure of soft interactions ($$p_{\mathrm {T}}$$$$<$$$$p_\mathrm{T0}$$), and one (ReconnectionProbability) giving the probability of CR without a $$p_{\mathrm {T}}$$ dependence for color strings. We include all four center-of-mass energies, although at each energy we exclude the first two $$p_\mathrm{T}^\mathrm{max}$$ bins. These first bins, e.g. for $$p_\mathrm{T}^\mathrm{max}<1.5\,\text {GeV} $$, are sensitive to single-diffraction dissociation, central-diffraction, and double-diffraction dissociation, but herwig++ contains only the ND component.

In Table 5, the parameters of the new CUETHppS1 are listed and compared to those from Tune UE-EE-5C. The parameters of the two tunes are very similar. The $\chi ^2$/dof, also indicated in Table 5, is found to be ${\approx } 0.46$, which is smaller than the value obtained for other CMS UE tunes. This is due to the fact that the first two bins as a function of $p_\mathrm{T}^\mathrm{max}$, which have much smaller statistical uncertainties than the higher-$p_\mathrm{T}^\mathrm{max}$ bins, are excluded from the fit because they cannot be described by any reasonable fit-values. In Appendix C, predictions obtained with herwig++ Tune UE-EE-5C and CUETHppS1 are compared to the UE data. The two tunes are both able to reproduce the UE data at all energies. With the new CUETHppS1 tune, uncertainties can be estimated using the eigentunes (Appendix A).

**Table 5:** The herwig++ parameters, tuning range, Tune UE-EE-5C values [30], and best-fit values for CUETHppS$1$, obtained from a fit to the TransMAX and TransMIN charged-particle and $p_\mathrm{T}^\mathrm{sum}$ densities as a function of the leading charged-particle $p_\mathrm{T}^\mathrm{max}$at $\sqrt{s} = 0.3$ , 0.9, 1.96, and $7\,\text {TeV}$

herwig++ Parameter	Tuning range	UE-EE-5C	CUETHppS1
PDF	–	CTEQ6L1	CTEQ6L1
MPIHandler:Power	0.1–0.5	0.33	0.371
RemnantDecayer:colourDisrupt	0.1–0.9	$0.8\phantom {0}$	0.628
MPIHandler:InvRadius [GeV$^2$]	0.5–2.7	2.30	2.255
ColourReconnector:ReconnectionProbability	0.1–0.9	0.49	0.528
MPIHandler:pTmin0 [GeV]	–	3.91	$3.91^\mathrm{a}$
MPIHandler:ReferenceScale [GeV]	–	7000	$7000^\mathrm{a}$
$\chi ^2$/dof	–	–	0.463

$^\mathrm{a}$ Fixed at Tune UE-EE-5C value

In conclusion, both herwig++ tunes, as well as the new CMS pythia6 UE tunes reproduce the UE data at all four $\sqrt{s}$. The pythia8 UE tunes, however, do not describe well the data at $\sqrt{s}=300\,\text {GeV}$, which may be related to the modelling of the proton–proton overlap function. The pythia6 Tune Z$2^*$lep, and the new CMS UE tunes use a double-Gaussian matter distribution, while all the pythia8 UE tunes use a single exponential matter overlap. The herwig++ tune, on the other hand, uses a matter-overlap function that is related to the Fourier transform of the electromagnetic form factor with $\mu ^2$ [7] playing the role of an effective inverse proton radius (i.e. the InvRadius parameter in Table 5). However, predictions from a tune performed with pythia8 using a double-Gaussian matter distribution were not able to improve the quality of the fit as a fit obtained without interleaved FSR in the simulation of the UE (as it is implemented in pythia6) did not show any improvement. Further investigations are needed to resolve this issue.

## The CMS DPS tunes

Traditionally, $$\sigma _\mathrm{eff}$$ is determined by fitting the DPS-sensitive observables with two templates [32–36] that are often based on distributions obtained from QCD MC models. One template is constructed with no DPS, i.e. just single parton scattering (SPS), while the other represents DPS production. This determines $$\sigma _\mathrm{eff}$$ from the relative amounts of SPS and DPS contributions needed to fit the data. Here we use an alternative method that does not require construction of templates from MC samples. Instead, we fit the DPS-sensitive observables directly and then calculate the resulting $$\sigma _\mathrm{eff}$$ from the model. For example, in pythia8, the value of $$\sigma _\mathrm{eff}$$ is calculated by multiplying the ND cross section by an enhancement or a depletion factor, which expresses the dependence of DPS events on the collision impact parameter. As expected, more central collisions have a higher probability of a second hard scattering than peripheral collisions. The enhancement/depletion factors depend on the UE parameters, namely, on the parameters that characterize the matter-overlap function of the two protons, which for bProfile$$=3$$ is determined by the exponential parameter expPow, on the MPI regulator $$p_\mathrm{T0}$$ in Eq. (2), and the range of the CR. pythia8 Tune $$4$$C gives $$\sigma _\mathrm{eff}$$$$\approx $$ 30.3 mb at $$\sqrt{s}=7\,\text {TeV} $$.

In Sect. 2, we determined the MPI parameters by fitting UE data. Here we determine the MPI parameters by fitting to observables which involve correlations among produced objects in hadron–hadron collisions that are sensitive to DPS. Two such observables used in the fit, $\varDelta S$ and $\varDelta ^\mathrm{rel}p_\mathrm{T}$, are defined as follows:

5 $$\begin{aligned}&\varDelta \mathrm{S}=\arccos \left( \frac{\vec {p}_{\text {T}}(\text {object}_1)\cdot \vec {p}_{\text {T}} (\text {object}_2)}{|\vec {p}_T(\text {object}_1)| \times |\vec {p}_{\text {T}}(\text {object}_2)|}\right) ,\end{aligned}$$

6 $$\begin{aligned}&\varDelta ^\mathrm{rel}p_\mathrm{T} = \frac{|\vec {p}_\mathrm{T}^{\ \mathrm jet_1}+\vec {p}_\mathrm{T}^{\ \mathrm jet_2}|}{|\vec {p}_\mathrm{T}^{\ \mathrm jet_1}|+|\vec {p}_\mathrm{T}^{\ \mathrm jet_2}|}, \end{aligned}$$

where, for $\mathrm {W}$+dijet production, object$_1$ is the $\mathrm {W}$ boson and object$_2$ is the dijet system. For four-jet production, object$_1$ is the hard-jet pair and object$_2$ is the soft-jet pair. For $\varDelta ^\mathrm{rel}p_\mathrm{T}$ in $\mathrm {W}$+dijet production, jet$_1$ and jet$_2$ are the two jets of the dijet system, while in four-jet production, jet$_1$ and jet$_2$ refer to the two softer jets.

The pythia8 UE parameters are fitted to the DPS-sensitive observables measured by CMS in $\mathrm {W}$+dijet [36] and in four-jet production [37]. After extracting the MPI parameters, the value of $\sigma _\mathrm{eff}$ in Eq. (3) can be calculated from the underlying MPI model. In pythia8, $\sigma _\mathrm{eff}$ depends primarily on the matter-overlap function and, to a lesser extent, on the value of $p_\mathrm{T0}$ in Eq. (2), and the range of the CR. We obtain two separate tunes for each channel: in the first one, we vary just the matter-overlap parameter expPow, to which the $\sigma _\mathrm{eff}$ value is most sensitive, and in the second one, the whole set of parameters is varied. These two tunes allow to check whether the value of $\sigma _\mathrm{eff}$ is stable relative to the choice of parameters.

The $\mathrm {W}$+dijet and the four-jet channels are fitted separately. The fit to DPS-sensitive observables in the $\mathrm {W}$+dijet channel gives a new determination of $\sigma _\mathrm{eff}$ which can be compared to the value measured through the template method in the same final state [36]. Fitting the same way to the observables in the four-jet final state provides an estimate of $\sigma _\mathrm{eff}$ for this channel.

### Double-parton scattering in W+dijet production

**Fig. 6:**
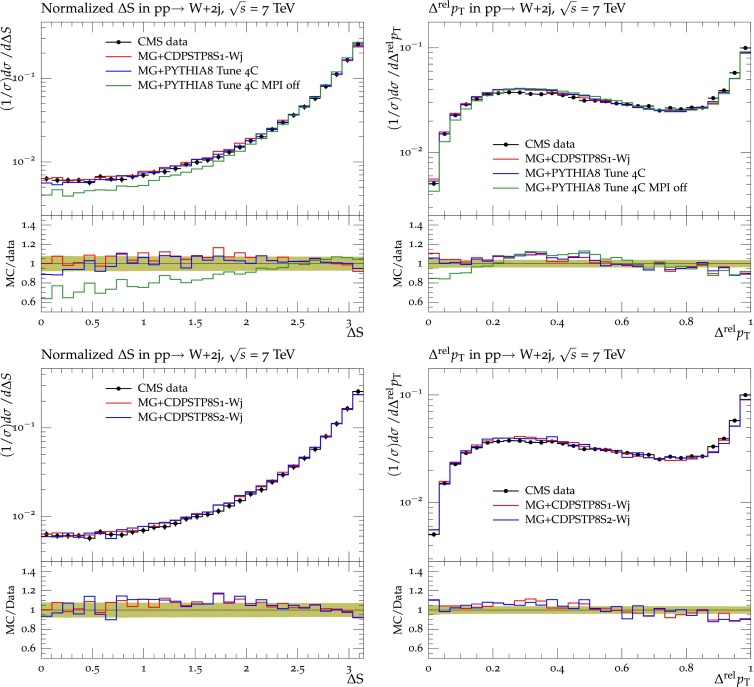
CMS data at $\sqrt{s}=7\,\text {TeV}$ [36] for the normalized distributions of the correlation observables $\varDelta$S (*left*), and $\varDelta ^\mathrm{rel}p_\mathrm{T}$ (*right*) in the $\mathrm {W}$+dijet channel, compared to MadGraph (MG) interfaced to: pythia8 Tune $4$C, Tune $4$C with no MPI, and the CMS pythia8 DPS partial CDPSTP$8$S$1$-Wj (*top*); and CDPSTP$8$S$1$-Wj, and CDPSTP$8$S$2$-Wj (*bottom*). The *bottom panels* of each plot show the ratios of these tunes to the data, and the *green bands* around unity represent the total experimental uncertainty

To study the dependence of the DPS-sensitive observables on MPI parameters, we construct two $$\mathrm {W}$$+dijet DPS tunes, starting from the parameters of pythia8 Tune $$4$$C. In a partial tune only the parameter of the exponential distribution expPow is varied, and in a full tune all four parameters in Table 6 are varied. In a comparison of models with $$\mathrm {W}$$+dijet events [36], it was shown that higher-order SPS contributions (not present in pythia) fill a similar region of phase-space as the DPS signal. When such higher-order SPS diagrams are neglected, the measured DPS contribution to the $$\mathrm {W}$$+dijet channel can be overestimated (i.e. $$\sigma _\mathrm{eff}$$ underestimated). We therefore interface the LO matrix elements (ME) generated by MadGraph 5 (version 1.5.14) [38] with pythia8, and tune to the normalized distributions of the correlation observables in Eqs. (5) and (6). For this study, we produce MadGraph parton-level events with a $$\mathrm {W}$$ boson and up to four partons in the final state. The cross section is calculated using the CTEQ6L1 PDF with a matching scale for ME and parton shower (PS) jets set to 20 GeV. (In Sect. 4, we show that the CMS UE tunes can be interfaced to higher-order ME generators without additional tuning of MPI parameters). Figure 6 shows the CMS data [36] for the observables $$\varDelta $$S and $$\varDelta ^\mathrm{rel}p_\mathrm{T}$$ measured in $$\mathrm {W}$$+dijet production, compared to predictions from MadGraph interfaced to pythia8 Tune $$4$$C, to Tune $$4$$C with no MPI, to the partial CDPSTP$$8$$S$$1$$-Wj, as well as to the full CDPSTP$$8$$S$$2$$-Wj (CDPST stands for “CMS DPS tune”). Table 6 gives the best-fit parameters and the resulting $$\sigma _\mathrm{eff}$$ values at $$\sqrt{s}=7\,\text {TeV} $$. The uncertainties quoted for $$\sigma _\mathrm{eff}$$ are computed from the uncertainties of the fitted parameters given by the eigentunes. For Tune $$4$$C, the uncertainty in $$\sigma _\mathrm{eff}$$ is not provided since no eigentunes are available for that tune. The resulting values of $$\sigma _\mathrm{eff}$$ are compatible with the value measured by CMS using the template method of $$\sigma _\mathrm{eff} = 20.6\pm 0.8 \,\text {(stat)} \pm 6.6 \,\text {(syst)} $$$$\text {\,mb}$$  [36].

**Table 6:** The pythia8 parameters, tuning ranges, Tune $4$C values [28] and best-fit values of CDPSTP$8$S$1$-Wj and CDPSTP$8$S$2$-Wj, obtained from fits to DPS observables in $\mathrm {W}$+dijet production with the MadGraph event generator interfaced to pythia8. Also shown are the predicted values of $\sigma _\mathrm{eff}$ at $\sqrt{s}=7\,\text {TeV}$, and the uncertainties obtained from the eigentunes

pythia8 Parameter	Tuning range	Tune $4$C	CDPSTP$8$S$1$-Wj	CDPSTP$8$S$2$-Wj
PDF		CTEQ6L1	CTEQ6L1	CTEQ6L1
MultipartonInteractions:pT0Ref [GeV]	1.0–3.0	2.085	$2.085^\mathrm{a}$	2.501
MultipartonInteractions:ecmPow	0.0–0.4	$0.19\phantom {0}$	$0.19^\mathrm{a}\phantom {0}$	0.179
MultipartonInteractions:expPow	0.4–10.0	$2.0\phantom {00}$	1.523	1.120
ColourReconnection:range	0.0–9.0	$1.5\phantom {00}$	$1.5^\mathrm{a}\phantom {0}$	2.586
MultipartonInteractions:ecmRef [GeV]	–	1800	$1800^\mathrm{a}$	$1800^\mathrm{a}$
$\chi ^2$/dof	–	–	0.118	0.09
Predicted $\sigma _\mathrm{eff}$ (in mb)	–	30.3	$25.9^{+2.4}_{-2.9}$	$25.8^{+8.2}_{-4.2}$

$^\mathrm{a}$ Fixed at Tune $4$C value

### Double-parton scattering in four-jet production

**Fig. 7:**
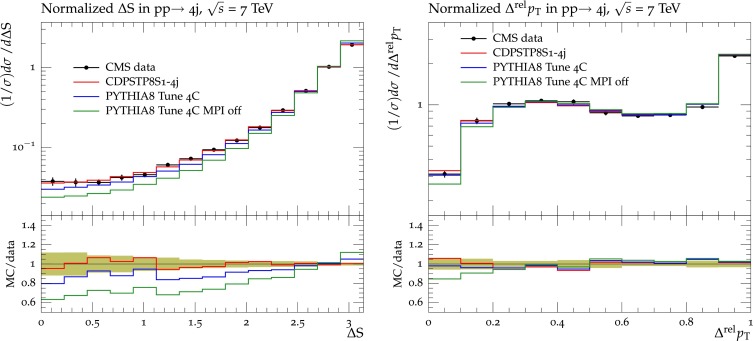
Distributions of the correlation observables $\varDelta$S (*left*) and $\varDelta ^\mathrm{rel}p_\mathrm{T}$ (*right*) measured in four-jet production at $\sqrt{s}=7\,\text {TeV}$ [37] compared to pythia8 Tune 4C, Tune 4C with no MPI, and CDPSTP8S1-4j. The *bottom panels* of each plot show the ratios of these predictions to the data, and the *green bands* around unity represent the total experimental uncertainty

**Fig. 8:**
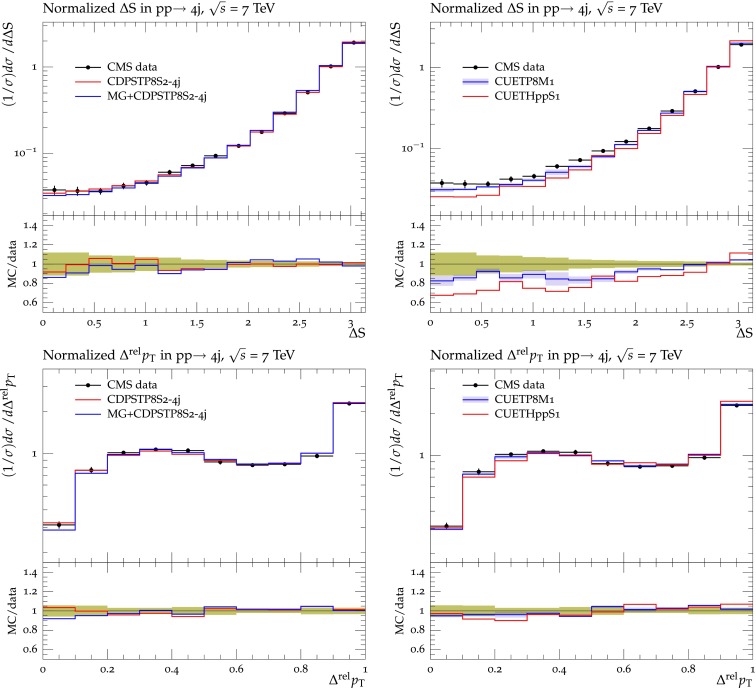
Distributions in the correlation observables $\varDelta$S (*top*) and $\varDelta ^\mathrm{rel}p_\mathrm{T}$ (*bottom*) measured in four-jet production at $\sqrt{s}=7\,\text {TeV}$ [37], compared to predictions of pythia8 using CDPSTP$8$S$2$-$4$j and of MadGraph (MG) interfaced to pythia8 using CDPSTP$8$S$2$-$4$j (*left*) and pythia8 using CUETP$8$M$1$ and herwig++ with CUETHppS$1$ (*right*). Also shown are the ratios of the predictions to the data. Predictions for CUETP$8$M$1$ (*right*) are shown with an error band corresponding to the total uncertainty obtained from the eigentunes (Appendix A). The *green bands* around unity represent the total experimental uncertainty

Starting from the parameters of pythia8 Tune $4$C, we construct two different four-jet DPS tunes. As in the $\mathrm {W}$+dijet channel, in the partial tune just the exponential-dependence parameter, expPow, while in the full tune all four parameters of Table 7 are varied. We obtain a good fit to the four-jet data without including higher-order ME contributions. However, we also obtain a good fit when higher-order (real) ME terms are generated with MadGraph. In Figs. 7 and 8 the correlation observables $\varDelta$S and $\varDelta ^\mathrm{rel}p_\mathrm{T}$ in four-jet production [37] are compared to predictions obtained with pythia8 Tune $4$C, Tune $4$C without MPI, CDPSTP$8$S$1$-$4$j, CDPSTP$8$S$2$-$4$j, and MadGraph interfaced to CDPSTP$8$S$2$-$4$j. Table 7 gives the best-fit parameters and the resulting $\sigma _\mathrm{eff}$ values. The values of $\sigma _\mathrm{eff}$ extracted from the CMS pythia8 DPS tunes give the first determination of $\sigma _\mathrm{eff}$ in four-jet production at $\sqrt{s}=7\,\text {TeV}$. The uncertainties quoted for $\sigma _\mathrm{eff}$ are obtained from the eigentunes.

**Table 7:** The pythia8 parameters, tuning ranges, Tune $4$C values [28] and best-fit values of CDPSTP$8$S$1$-$4$j and CDPSTP$8$S$2$-$4$j, obtained from fits to DPS observables in four-jet production. Also shown are the predicted values of $\sigma _\mathrm{eff}$ at $\sqrt{s}=7\,\text {TeV}$, and the uncertainties obtained from the eigentunes

pythia8 Parameter	Tuning range	Tune $4$C	CDPSTP$8$S$1$-$4$j	CDPSTP$8$S$2$-$4$j
PDF		CTEQ6L1	CTEQ6L1	CTEQ6L1
MultipartonInteractions:pT0Ref [GeV]	1.0–3.0	2.085	$2.085^\mathrm{a}$	2.125
MultipartonInteractions:ecmPow	0.0–0.4	$0.19\phantom {0}$	$0.19^\mathrm{a}\phantom {0}$	0.179
MultipartonInteractions:expPow	$\phantom {0}0.4$–10.0	$2.0\phantom {00}$	$1.160\phantom {0}$	0.692
ColourReconnection:range	0.0–9.0	$1.5\phantom {00}$	$1.5^\mathrm{a}\phantom {00}$	6.526
MultipartonInteractions:ecmRef [GeV]	–	1800	$1800^\mathrm{a}$	$1800^\mathrm{a}$
$\chi ^2$/dof	–	–	0.751	0.428
Predicted $\sigma _\mathrm{eff}$ (in mb)	–	30.3	$21.3^{+1.2}_{-1.6}$	$19.0^{+4.7}_{-3.0}$

$^\mathrm{a}$ Fixed at Tune $4$C value

## Validation of CMS tunes

Here we discuss the compatibility of the UE and DPS tunes. In addition, we compare the CMS UE tunes with UE data that have not been used in the fits, and we examine how well Drell–Yan and MB observables can be predicted from MC simulations using the UE tunes. We also show that the CMS UE tunes can be interfaced to higher-order ME generators without additional tuning of the MPI parameters.

### Compatibility of UE and DPS tunes

**Fig. 9:**
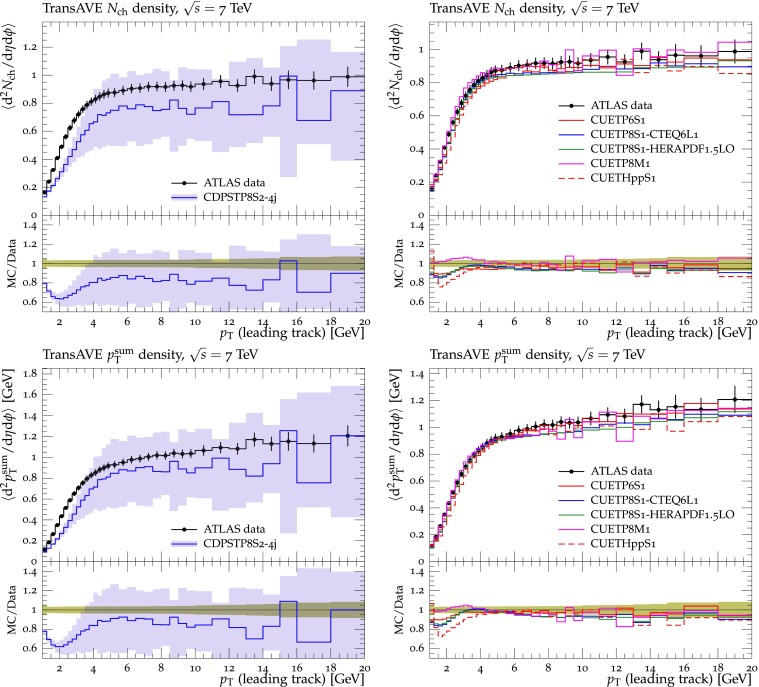
ATLAS data at $\sqrt{s}=7\,\text {TeV}$ [39] for charged-particle (*left*) and $p_\mathrm{T}^\mathrm{sum}$ densities (*right*) with $p_\mathrm{T}\!>\!0.5\,\text {GeV}$ and $|\eta |\!<\!2.0$ in the transverse (TransAVE) region compared to predictions of pythia8 using CDPSTP$8$S$2$-$4$j (*left*) and CUETP$8$S$1$-CTEQ$6$L1, CUETP$8$S$1$-HERAPDF1.5LO, and CUETP$8$M$1$, plus herwig++ using CUETHppS$1$ (*right*). The predictions of CDPSTP$8$S$2$-$4$j are shown with an error band corresponding to the total uncertainty obtained from the eigentunes (Appendix A). The *bottom panels* of each plot show the ratios of these predictions to the data, and the *green bands* around unity represent the total experimental uncertainty

The values of $$\sigma _\mathrm{eff}$$ obtained from simulations applying the CMS pythia8 UE and DPS tunes at $$\sqrt{s}=7\,\text {TeV} $$ and $$\sqrt{s}=13\,\text {TeV} $$ are listed in Table 8. The uncertainties, obtained from eigentunes are also quoted in Table 8. At $$\sqrt{s}=7\,\text {TeV} $$, the CMS DPS tunes give values of $$\sigma _\mathrm{eff}$$$$\approx $$ 20$$\text {\,mb}$$, while the CMS pythia8 UE tunes give slightly higher values in the range 26–29 mb as shown in Figs. 8 and  9. Figure 8 shows the CMS DPS-sensitive data for four-jet production at $$\sqrt{s}=7\,\text {TeV} $$ compared to predictions using CDPSTP$$8$$S$$2$$-$$4$$j, CUETP$$8$$M$$1$$, and CUETHppS$$1$$. Figure 9 shows ATLAS UE data at $$\sqrt{s}=7\,\text {TeV} $$ [39] compared to predictions obtained with various tunes: CDPSTP$$8$$S$$2$$-$$4$$j with uncertainty bands, CUETP$$6$$S$$1$$-CTEQ$$6$$L1, CUETP$$8$$S$$1$$-CTEQ$$6$$L1, CUETP$$8$$S$$1$$-HERAPDF1.5LO, CUETP$$8$$M$$1$$, and CUETHppS$$1$$. Predictions from pythia8 using CUETP$$8$$M$$1$$ describe reasonably well the DPS observables, but do not fit them as well as predictions using the DPS tunes. On the other hand, predictions using CDPSTP$$8$$S$$2$$-$$4$$j do not fit the UE data as well as the UE tunes do.

**Table 8:** Values of $\sigma _\mathrm{eff}$ at $\sqrt{s}=7\,\text {TeV}$ and $13\,\text {TeV}$ for CUETP$8$S$1$-CTEQ$6$L1, CUETP$8$S$1$-HERAPDF1.5LO, and CUETP$8$M$1$, CUETHppS$1$, and for CDPSTP$8$S$1$-$4$j and CDPSTP$8$S$2$-$4$j. At $\sqrt{s}=7\,\text {TeV}$, also shown are the uncertainties in $\sigma _\mathrm{eff}$ obtained from the eigentunes

CMS tune	$\sigma _\mathrm{eff}$(mb) at $7\,\text {TeV}$	$\sigma _\mathrm{eff}$(mb) at $13\,\text {TeV}$
CUETP$8$S$1$-CTEQ$6$L1	$27.8^{+1.2}_{-1.3}$	$29.9^{+1.6}_{-2.8}$
CUETP$8$S$1$-HERAPDF1.5LO	$29.1^{+2.2}_{-2.0}$	$31.0^{+3.8}_{-2.6}$
CUETP$8$M$1$	$26.0^{+0.6}_{-0.2}$	$27.9^{+0.7}_{-0.4}$
CUETHppS$1$	$15.2^{+0.5}_{-0.6}$	$15.2^{+0.5}_{-0.6}$
CDPSTP$8$S$1$-$4$j	$21.3^{+1.2}_{-1.6}$	$21.8^{+1.0}_{-0.7}$
CDPSTP$8$S$2$-$4$j	$19.0^{+4.7}_{-3.0}$	$22.7^{+10.0}_{-5.2}$

As discussed previously, the pythia8 tunes use a single exponential matter-overlap function, while the herwig++ tune uses a matter-overlap function that is related to the Fourier transform of the electromagnetic form factor. The CUETHppS$$1$$ gives a value of $$\sigma _\mathrm{eff}$$$$\approx $$ 15$$\text {\,mb}$$, while UE and DPS tunes give higher values of $$\sigma _\mathrm{eff}$$. It should be noted that $$\sigma _\mathrm{eff}$$ is a parton-level observable and its importance is not in the modelled value of $$\sigma _\mathrm{eff}$$, but in what is learned about the transverse proton profile (and its energy evolution), and how well the models describe the DPS-sensitive observables. As can be seen in Fig. 8, predictions using CUETP$$8$$M$$1$$ describe the DPS-sensitive observables better than CUETHppS$$1$$, but not quite as well as the DPS tunes. We performed a simultaneous pythia8 tune that included both the UE data and DPS-sensitive observables, however, the quality of the resulting fit was poor. This confirms the difficulty of describing soft and hard MPI within the current pythia and herwig++ frameworks. Recent studies [40, 41] suggest the need for introducing parton correlation effects in the MPI framework in order to achieve a consistent description of both the UE and DPS observables.

### Comparisons with other UE measurements

**Fig. 10:**
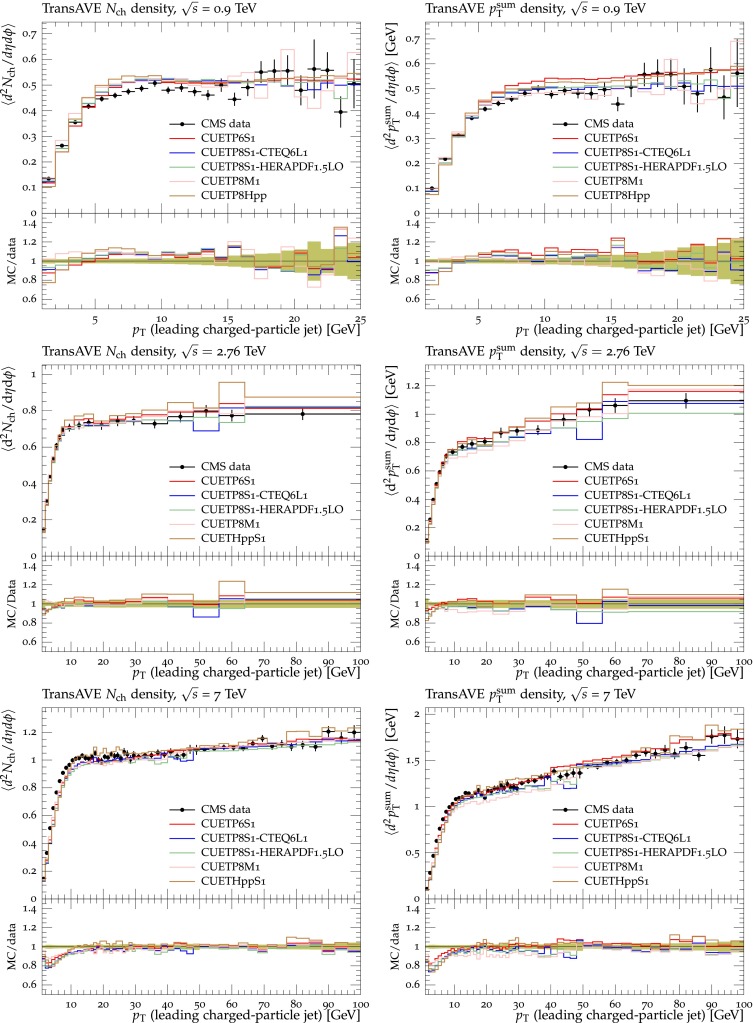
CMS data on charged-particle (*left*) and $p_\mathrm{T}^\mathrm{sum}$ (*right*) densities at $\sqrt{s}$ = 0.9 [24] (*top*), 2.76 [42] (*middle*), and $7\,\text {TeV}$ [24] (*bottom*) with $p_\mathrm{T}\!>\!0.5\,\text {GeV}$ and $|\eta |\!<\!2.0$ in the transverse (TransAVE) region as defined by the leading charged-particle jet, as a function of the transverse momentum of the leading charged-particle jet. The data are compared to predictions of pythia6 using CUETP$6$S$1$-CTEQ$6$L1, pythia8 using CUETP$8$S$1$-CTEQ$6$L1, CUETP$8$S$1$-HERAPDF1.5LO, and CUETP$8$M$1$, and herwig++ using CUETHppS$1$. The *bottom panels* of each plot show the ratios of these predictions to the data, and the *green bands* around unity represent the total experimental uncertainty

Figure 10 shows charged particle and $p_\mathrm{T}^\mathrm{sum}$ densities [24, 42] at $\sqrt{s} = 0.9$, 2.76, and $7\,\text {TeV}$ with $p_\mathrm{T}\!>\!0.5\,\text {GeV}$ and $|\eta |\!<\!2.0$ in the TransAVE region, as defined by the leading jet reconstructed by using just the charged particles (also called “leading track-jet”) compared to predictions using the CMS UE tunes. The CMS UE tunes describe quite well the UE measured using the leading charged particle as well as the leading charged-particle jet.

**Fig. 11:**
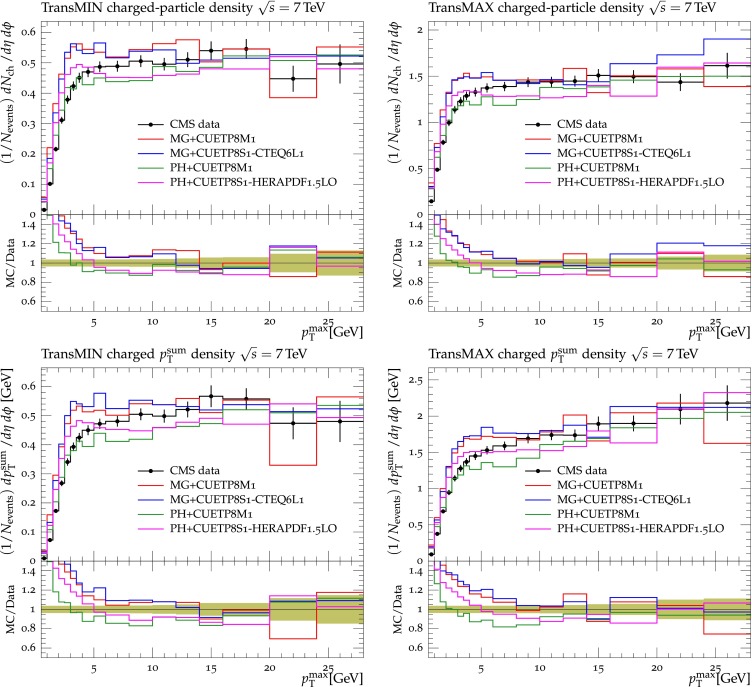
CMS data at $\sqrt{s}=7\,\text {TeV}$  [17] for particle (*top*) and $p_\mathrm{T}^\mathrm{sum}$ densities (*bottom*) for charged particles with $p_\mathrm{T}\!>\!0.5\,\text {GeV}$ and $|\eta |\!<\!0.8$ in the TransMIN (*left*) and TransMAX (*right*) regions, as defined by the leading charged particle, as a function of the transverse momentum of the leading charged-particle $p_\mathrm{T}^\mathrm{max}$. The data are compared to MadGraph (MG), interfaced to pythia8 using CUETP$8$S$1$-CTEQ$6$L1 and CUETP$8$M$1$, and to powheg (PH), interfaced to pythia8 using CUETP$8$S$1$-HERAPDF1.5LO and CUETP$8$M$1$. The *bottom panels* of each plot show the ratios of these predictions to the data, and the *green bands* around unity represent the total experimental uncertainty

Tunes obtained from fits to UE data and combined with higher-order ME calculations [43] can also be cross-checked against the data. The CMS UE tunes can be interfaced to higher-order ME generators without spoiling their good description of the UE. In Fig. 11, the charged-particle and $p_\mathrm{T}^\mathrm{sum}$ densities in the TransMIN and TransMAX regions as a function of $p_\mathrm{T}^\mathrm{max}$, are compared to predictions obtained with MadGraph and powheg  [44, 45] interfaced to pythia8 using CUETP$8$S$1$-CTEQ$6$L1 and CUETP$8$M$1$. In MadGraph, up to four partons are simulated in the final state. The cross section is calculated with the CTEQ6L1 PDF. The ME/PS matching scale is taken to be 10$\,\text {GeV}$. The powheg predictions are based on next-to-leading-order (NLO) dijet using the CT10nlo PDF [46] interfaced to pythia8 based on CUETP$8$M$1$, and HERAPDF1.5NLO [21] interfaced to the pythia8 using CUETP$8$S$1$-HERAPDF1.5LO.

The poor agreement below $$p_\mathrm{T}^\mathrm{max}$$$$=5\,\text {GeV} $$ in Fig. 11 is not relevant as the minimum $$\hat{p}_\mathrm{T}$$ for MadGraph and powheg is $$5\,\text {GeV} $$. The agreement with the UE data in the plateau region of $$p_\mathrm{T}^\mathrm{max}$$$$> 5\,\text {GeV} $$ is good. All these figures show that CMS UE tunes interfaced to higher-order ME generators do not spoil their good description of the UE data.

### Predicting MB observables

**Fig. 12:**
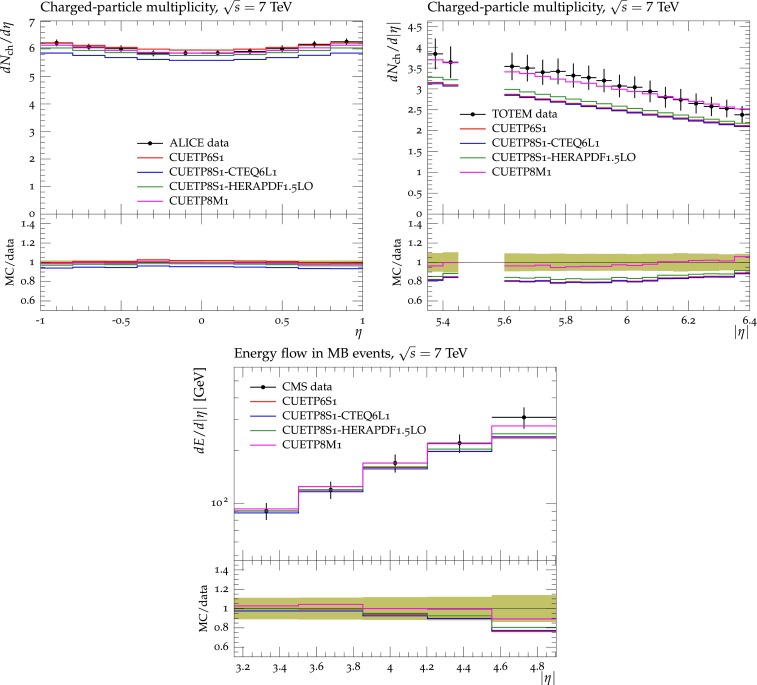
ALICE data at $\sqrt{s}=7\,\text {TeV}$ [47] for the charged-particle pseudorapidity distribution, $\mathrm{d}{\mathrm {N}}_{\text {ch}}/ \mathrm{d}\eta$, in inclusive inelastic $\mathrm {p}\mathrm {p}$ collisions (*top left*). TOTEM data at $\sqrt{s}=7\,\text {TeV}$ [48] for the charged-particle pseudorapidity distribution, $\mathrm{d}{\mathrm {N}}_{\text {ch}}/ \mathrm{d}\eta$, in inclusive inelastic pp collisions ($p_\mathrm{T}>40\,\text {MeV}$, ${\mathrm {N}}_\mathrm{chg}\ge 1$) (*top right*). CMS data at $\sqrt{s}=7\,\text {TeV}$ [50] for the energy flow $\mathrm{d}E/ \mathrm{d}\eta$, in MB $\mathrm {p}\mathrm {p}$ collisions. The data are compared to pythia6 using CUETP$6$S$1$-CTEQ$6$L1, and to pythia8 using CUETP$8$S$1$-CTEQ$6$L1, CUETP$8$S$1$-HERAPDF1.5LO, and CUETP$8$M$1$. The *bottom panels* of each plot show the ratios of these predictions to the data, and the *green bands* around unity represent the total experimental uncertainty

**Fig. 13:**
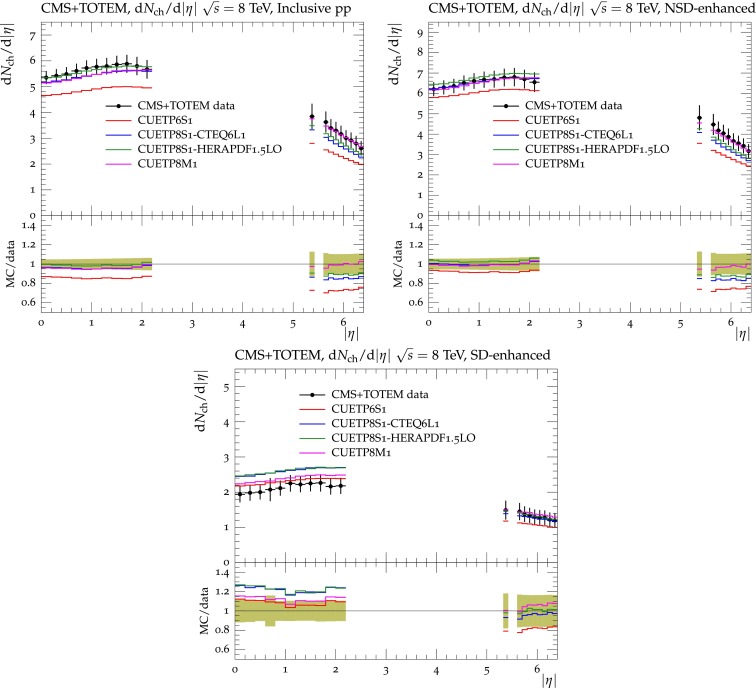
Combined CMS and TOTEM data at $\sqrt{s}=8\,\text {TeV}$ [50] for the charged-particle distribution $\mathrm{d}{\mathrm {N}}_{\text {ch}}/ \mathrm{d}\eta$, in inclusive inelastic (*top left*), NSD-enhanced (*top right*), and SD-enhanced (*bottom*) pp collisions. The data are compared to pythia6 using CUETP$6$S$1$-CTEQ$6$L1, and to pythia8 using CUETP$8$S$1$-CTEQ$6$L1, CUETP$8$S$1$-HERAPDF1.5LO, and CUETP$8$M$1$. The *bottom panels* of each plot show the ratios of these predictions to the data, and the *green bands* around unity represent the total experimental uncertainty

The UE is studied in events containing a hard scatter, whereas most of the MB collisions are softer and can include diffractive scatterings. It is however interesting to see how well predictions based on the CMS UE tunes can describe the properties of MB distributions. Figure 12 shows predictions using CMS UE tunes for the ALICE [47] and TOTEM data [48] at $\sqrt{s}=7\,\text {TeV}$ for the charged-particle pseudorapidity distribution, $\mathrm{d}{\mathrm {N}}_{\text {ch}}/\mathrm{d}\eta$, and for $\mathrm{d}E/\mathrm{d}\eta$ [49] at $\sqrt{s}=7\,\text {TeV}$. These observables are sensitive to single-diffraction dissociation, central-diffraction, and double-diffraction dissociation, which are modelled in pythia. Since herwig++ does not include a model for single-diffraction dissociation, central-diffraction, and double-diffraction dissociation, we do not show it here. Figure 13 shows predictions using the CMS UE tunes for the combined CMS$+$TOTEM data at $\sqrt{s}=8\,\text {TeV}$ [50] for the charged-particle pseudorapidity distribution, $\mathrm{d}{\mathrm {N}}_{\text {ch}}/\mathrm{d}\eta$, for inelastic, non single-diffraction-enhanced, and single-diffraction-enhanced proton–proton collisions.

**Fig. 14:**
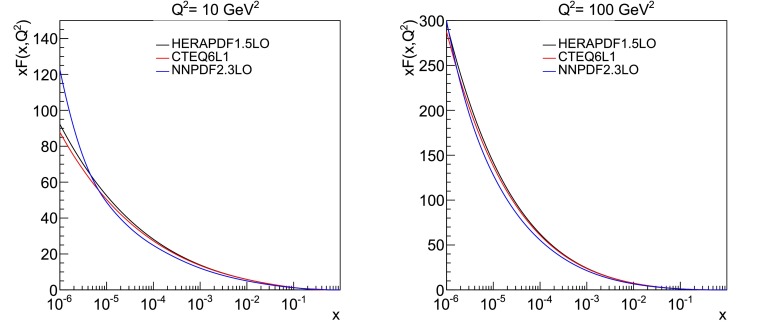
Comparison of gluon distributions in the proton for the CTEQ6L1, HERAPDF1.5LO, and NNPDF2.3LO PDF sets, at the $Q^2$ = 10 GeV$^2$ (*left*) and 100 GeV$^2$ (*right*)

The pythia8 event generator using the UE tunes describes the MB data better than pythia6 with the UE tune, which is likely due to the improved modelling of single-diffraction dissociation, central-diffraction, and double-diffraction dissociation in pythia8. Predictions with all the UE tunes describe fairly well MB observables in the central region ($$|\eta |<2$$), however, only predictions obtained with CUETP$$8$$M$$1$$ describe the data in the forward region ($$|\eta |>4$$). This is due to the PDF used in CUETP$$8$$M$$1$$. As can be seen in Fig. 14, the NNPDF2.3LO PDF at scales $$Q^2$$ = 10 GeV$$^2$$ (corresponding to hard scatterings with $$\hat{p}_\mathrm{T}$$$$\sim $$ 3 GeV) and small *x*, features a larger gluon density than in CTEQ6L1 and HERAPDF$$1.5$$LO, thereby contributing to more particles (and more energy) produced in the forward region. We have checked that increasing the gluon distribution in HERAPDF$$1.5$$LO at values below 10$$^{-5}$$ improved the description of the charged-particle multiplicity measurements in the forward region.

### Comparisons with inclusive jet production

**Fig. 15:**
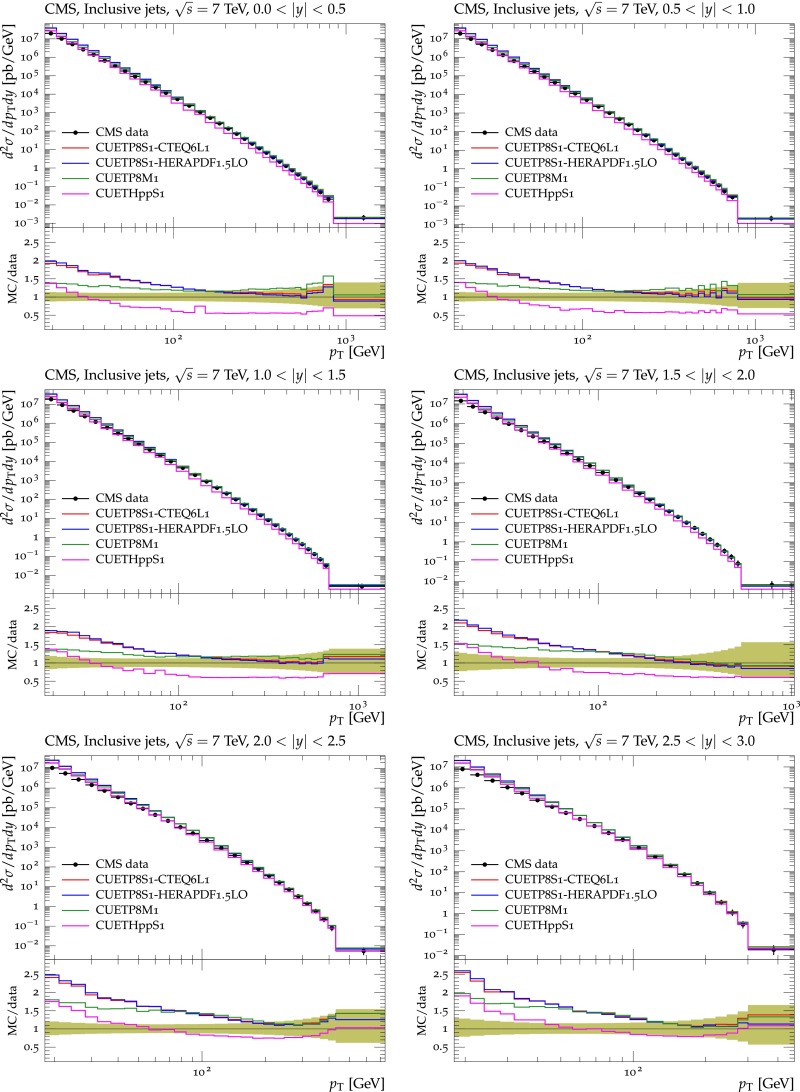
CMS data at $\sqrt{s}=7\,\text {TeV}$ [51] for the inclusive jet cross section as a function of $p_{\mathrm {T}}$ in different rapidity ranges compared to predictions of pythia8 using CUETP8S1-CTEQ6L1, CUETP8S1-HERAPDF, and CUETP8M1, and of herwig++ using CUETHppS1. The *bottom panels* of each plot show the ratios of these predictions to the data, and the *green bands* around unity represent the total experimental uncertainty

**Fig. 16:**
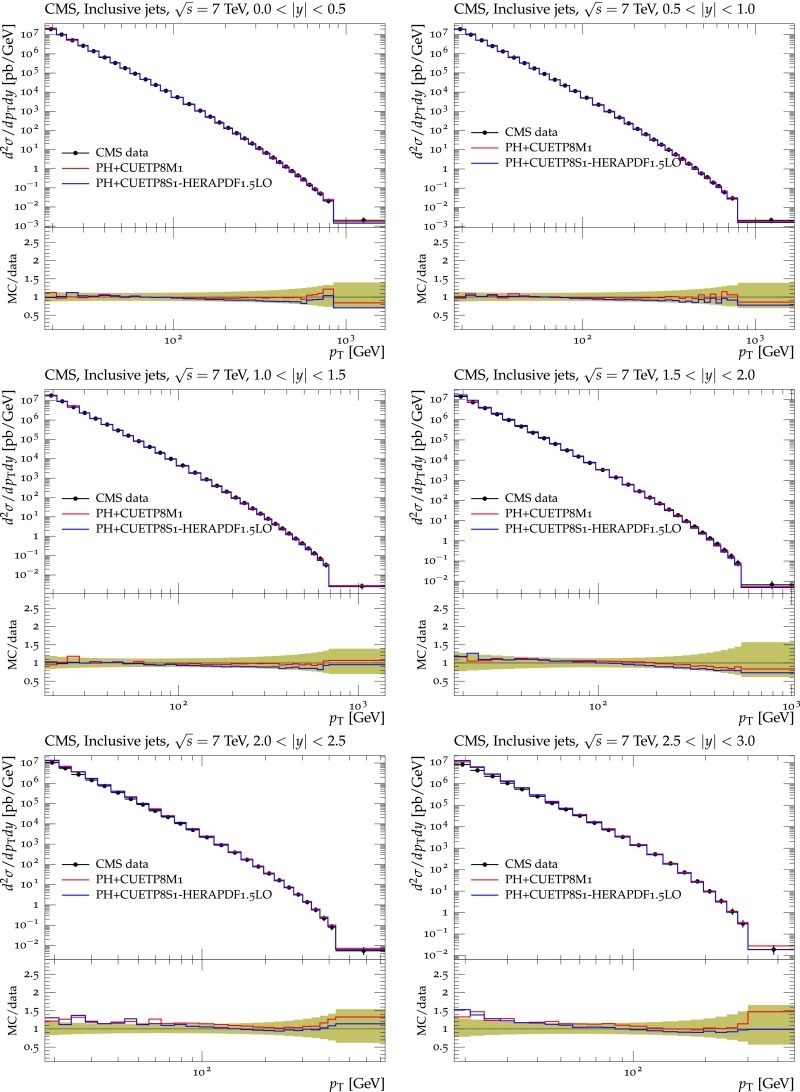
CMS data at $\sqrt{s}=7\,\text {TeV}$ [51] for the inclusive jet cross section as a function of $p_{\mathrm {T}}$ in different rapidity ranges compared to predictions of powheg interfaced to pythia8 using CUETP$8$S$1$-HERAPDF1.5LO and CUETP$8$M$1$. The *bottom panels* of each plot show the ratios of these predictions to the data, and the *green bands* around unity represent the total experimental uncertainty

In Fig. 15 predictions using CUETP$8$S$1$-CTEQ$6$L1, CUETP$8$S$1$-HERAPDF1.5LO, and CUETP$8$M$1$, and CUETHppS1 are compared to inclusive jet cross section at $\sqrt{s}=7\,\text {TeV}$ [51] in several rapidity ranges. Predictions using CUETP$8$M$1$ describe the data best, however, all the tunes overshoot the jet spectra at small $p_{\mathrm {T}}$. Predictions from the CUETHppS1 underestimate the high $p_\mathrm{T}$ region at central rapidity (|*y*| $<$ 2.0). In Fig. 16, the inclusive jet cross sections are compared to predictions from powheg interfaced to pythia8 using CUETP$8$S$1$-HERAPDF1.5LO and CUETP$8$M$1$. A very good description of the measurement is obtained.

### Comparisons with Z boson production

**Fig. 17:**
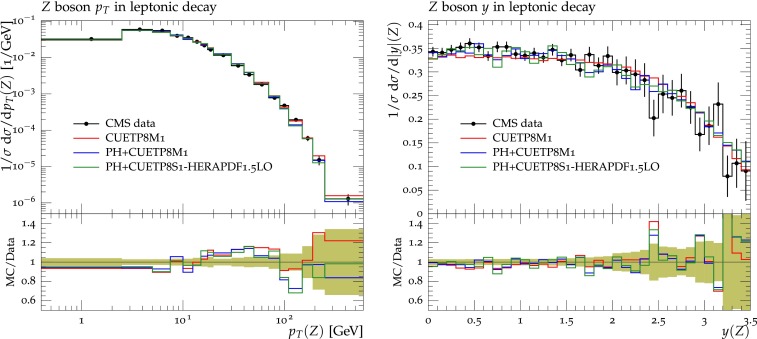
Transverse momentum $p_{\mathrm {T}}$ (*left*) and rapidity distributions (*right*) of $\mathrm{Z}$ boson production in pp collisions at $\sqrt{s}=7\,\text {TeV}$ [52]. The data are compared to pythia8 using CUETP$8$M$1$, and to powheg interfaced to pythia8 using CUETP$8$S$1$-CTEQ$6$L1 and CUETP$8$M$1$. The *green bands* in the ratios represent the total experimental uncertainty

In Fig. 17 the $p_{\mathrm {T}}$ and rapidity distributions of the $\mathrm{Z}$ boson in pp collisions at $\sqrt{s}=7\,\text {TeV}$ [52] are shown and compared to pythia8 using CUETP$8$M$1$, and to powheg interfaced to pythia8 using CUETP$8$S$1$-CTEQ$6$L1 and CUETP$8$M$1$. The prediction using pythia8 with CUETP$8$M$1$ (without powheg) agrees reasonably well with the distribution of the $\mathrm{Z}$ boson at small $p_{\mathrm {T}}$ values. Also, when interfaced to powheg, which implements an inclusive $\mathrm{Z}$ boson NLO calculation, the agreement is good over the whole spectrum.

**Fig. 18:**
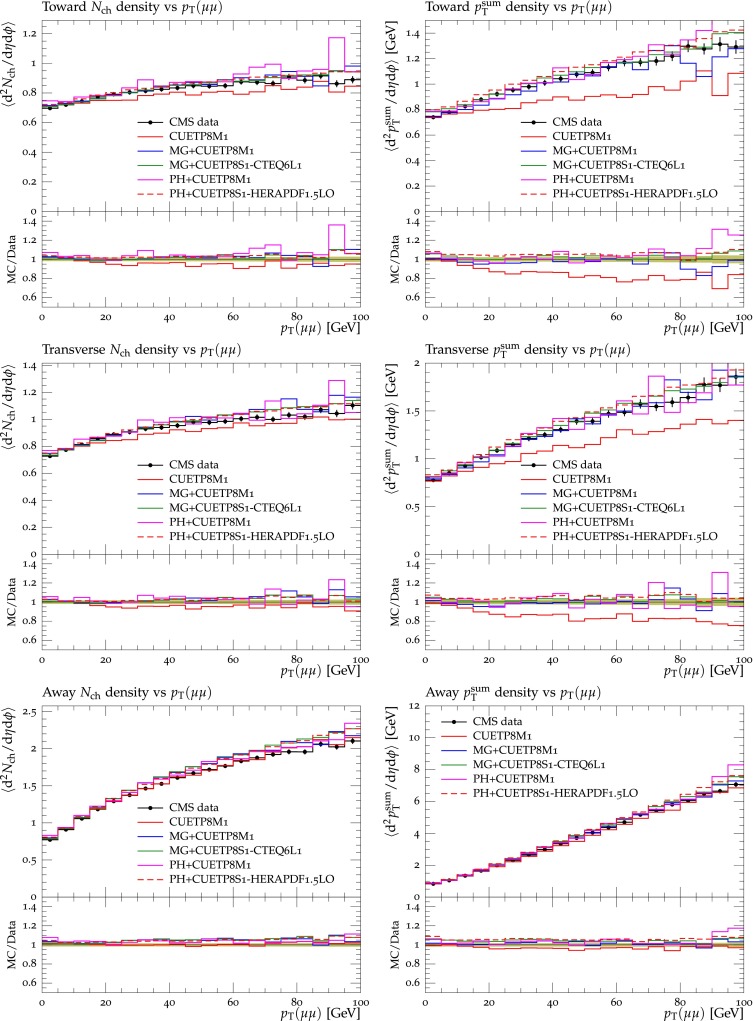
Charged-particle (*left*) and $p_\mathrm{T}^\mathrm{sum}$ densities (*right*) in the toward (*top*), away (*middle*), and transverse (TransAVE) (*bottom*) regions, as defined by the Z-boson direction in Drell–Yan production at $\sqrt{s}=7\,\text {TeV}$ [26]. The data are compared to pythia8 using CUETP$8$M$1$, to MadGraph (MG) interfaced to pythia8 using CUETP$8$S$1$-CTEQ$6$L1 and CUETP$8$M$1$, and to powheg (PH) interfaced to pythia8 using CUETP$8$S$1$-HERAPDF1.5LO and CUETP$8$M$1$. The *green bands* in the ratios represent the total experimental uncertainty

In Fig. 18 the charged-particle and $p_\mathrm{T}^\mathrm{sum}$ densities [26] in the toward, away, and transverse (TransAVE) regions as defined by the $\mathrm{Z}$ boson in proton–proton collisions at $\sqrt{s}=7\,\text {TeV}$ are compared to predictions of pythia8 using CUETP$8$M$1$. Also shown are MadGraph and powheg results interfaced to pythia8 using CUETP$8$S$1$-HERAPDF1.5LO and CUETP$8$M$1$. The MadGraph generator simulates Drell–Yan events with up to four partons, using the CTEQ6L1 PDF. The matching of ME partons and PS is performed at a scale of 20 GeV. The powheg events are obtained using NLO inclusive Drell–Yan production, including up to one additional parton. The powheg events are interfaced to pythia8 using CUETP$8$M$1$ and CUETP$8$S$1$-HERAPDF1.5LO. The predictions based on CUETP$8$M$1$ do not fit the $\mathrm{Z}$ boson data unless they are interfaced to a higher-order ME generator. In pythia8 only the Born term ($\mathrm{q} \overline{\mathrm{q}} \rightarrow \mathrm{Z}$), corrected for single-parton emission, is generated. This ME configuration agrees well with the observables in the away region in data, when the $\mathrm{Z}$ boson recoils against one or more jets. In the transverse and toward regions, larger discrepancies between data and pythia8 predictions appear at high $p_{\mathrm {T}}$, where the occurrence of multijet emission has a large impact. To describe $\mathrm{Z}$ boson production at $\sqrt{s}=7\,\text {TeV}$ in all regions, higher-order contributions (starting with $\mathrm{Z}$+2-jets), as used in interfacing pythia to powheg or MadGraph, must be included.

## Extrapolation to 13 TeV

**Fig. 19:**
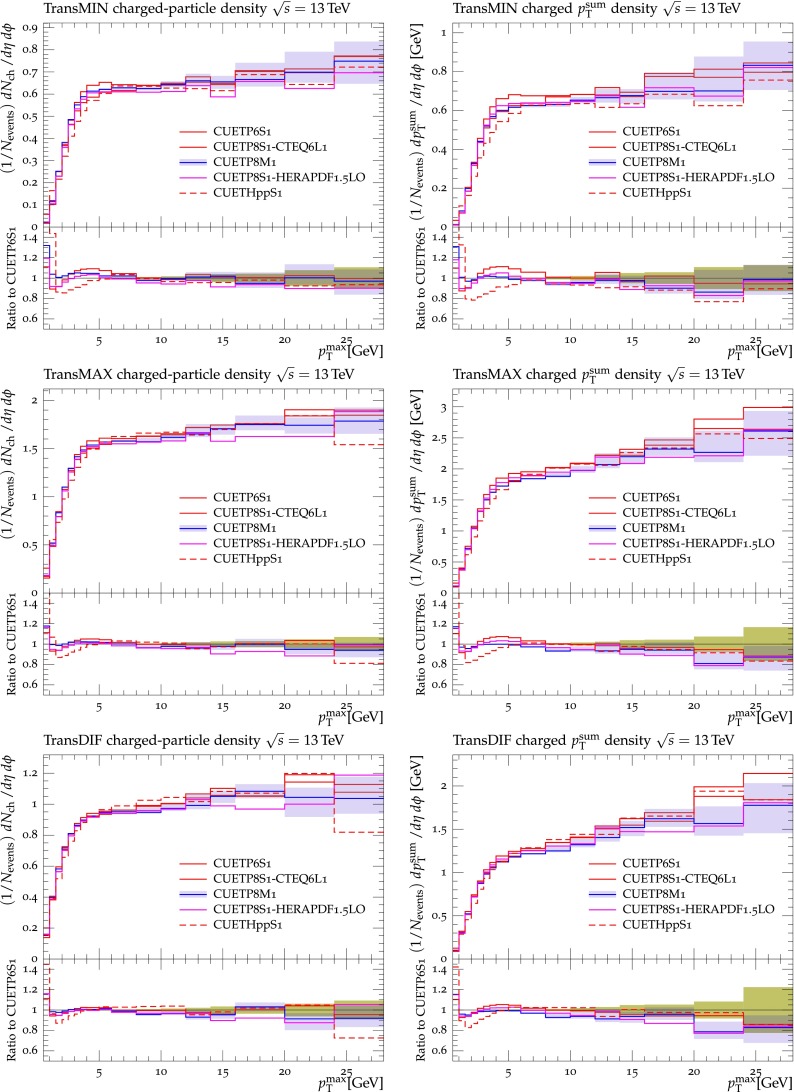
Predictions at $\sqrt{s}=13\,\text {TeV}$ for the particle (*left*) and the $p_\mathrm{T}^\mathrm{sum}$ densities (*right*) for charged particles with $p_\mathrm{T}\!>\!0.5\,\text {GeV}$ and $|\eta |\!<\!0.8$ in the TransMIN (*top*), TransMAX (*middle*), and TransDIF (*bottom*) regions, as defined by the leading charged particle, as a function of the leading charged-particle $p_\mathrm{T}^\mathrm{max}$ for the five CMS UE tunes: pythia6 CUETP$6$S$1$-CTEQ$6$L1, and pythia8 CUETP$8$S$1$-CTEQ$6$L1, CUETP$8$S$1$-HERAPDF1.5LO, and CUETP$8$M$1$, and herwig++ CUETHppS$1$. Also shown are the ratio of the tunes to predictions of CUETP$8$S$1$-CTEQ$6$L1. Predictions for CUETP$8$M$1$ are shown along with the envelope (*green bands*) of the corresponding eigentunes

In this section, predictions at $\sqrt{s}=13\,\text {TeV}$, based on the new tunes, for observables sensitive to the UE are presented. Figure 19 shows the predictions at 13 TeV for the charged-particle and the $p_\mathrm{T}^\mathrm{sum}$ densities in the TransMIN, TransMAX, and TransDIF regions, as defined by the leading charged particle as a function of $p_\mathrm{T}^\mathrm{max}$ based on the five new CMS UE tunes: CUETP$6$S$1$-CTEQ$6$L1, CUETP$8$S$1$-CTEQ$6$L1, CUETP$8$S$1$-HERAPDF1.5LO, CUETP$8$M$1$, and CUETHppS$1$. In Fig. 19 the ratio of the predictions using the four CMS tunes to the one using CUETP$8$M$1$ is shown. The predictions at $13\,\text {TeV}$ of all these tunes are remarkably similar. It does not seem to matter that the new CMS pythia8 UE tunes do not fit very well to the $\sqrt{s}=300\,\text {GeV}$ UE data. The new pythia8 tunes give results at $13\,\text {TeV}$ similar to the new CMS pythia6 tune and the new CMS herwig++ tune. The uncertainties on the predictions based on the eigentunes do not exceed 10 % relative to the central value.

**Fig. 20:**
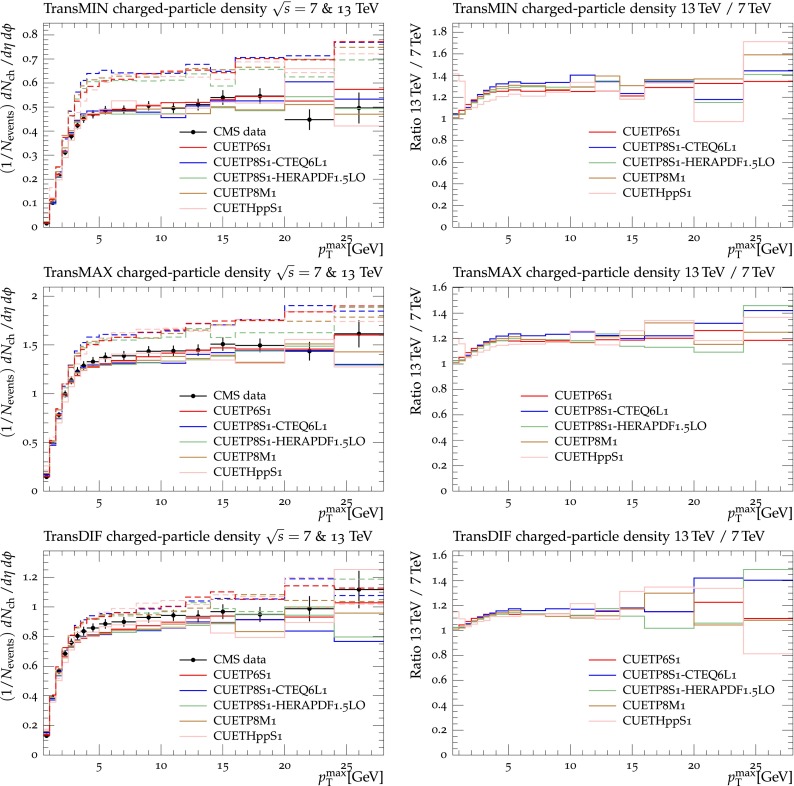
Charged-particle density at $\sqrt{s}=7\,\text {TeV}$ for particles with $p_\mathrm{T}\!>\!0.5\,\text {GeV}$ and $|\eta |\!<\!0.8$ in the TransMIN (*top*), TransMAX (*middle*), and TransDIF (*bottom*) regions, as defined by the leading charged particle, as a function of the leading charged-particle $p_\mathrm{T}^\mathrm{max}$. The data are compared to pythia6 using CUETP$6$S$1$-CTEQ$6$L1, to pythia8 using CUETP$8$S$1$-CTEQ$6$L1, CUETP$8$S$1$-HERAPDF1.5LO, and CUETP$8$M$1$, and to herwig++ using CUETHppS$1$. Also shown are the predictions (*left*) based on the CMS UE tunes at $13\,\text {TeV}$ (*dashed lines*), and the ratio of the $13\,\text {TeV}$ to $7\,\text {TeV}$ results for the five tunes (*right*)

**Fig. 21:**
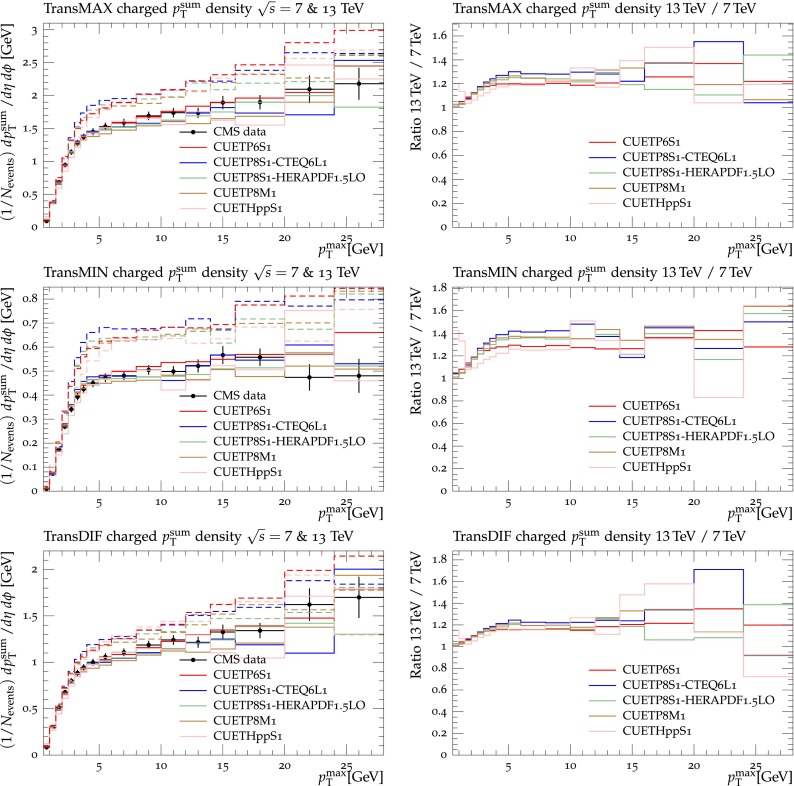
Charged $p_\mathrm{T}^\mathrm{sum}$ density at $\sqrt{s}=7\,\text {TeV}$ for particles with $p_\mathrm{T}\!>\!0.5\,\text {GeV}$ and $|\eta |\!<\!0.8$ in the TransMIN (*top*), TransMAX (*middle*), and TransDIF (*bottom*) regions, as defined by the leading charged particle, as a function of the leading charged-particle $p_\mathrm{T}^\mathrm{max}$. The data are compared to pythia6 using CUETP$6$S$1$-CTEQ$6$L1, to pythia8 using CUETP$8$S$1$-CTEQ$6$L1, CUETP$8$S$1$-HERAPDF1.5LO, and CUETP$8$M$1$, and to herwig++ using CUETHppS$1$. Also shown are the predictions (*left*) based on the CMS UE tunes at $13\,\text {TeV}$ (*dashed lines*), and the ratio of the $13\,\text {TeV}$ to $7\,\text {TeV}$ results for the five tunes (*right*)

In Figs. 20 and 21 the predictions at $$\sqrt{s}=13\,\text {TeV} $$ obtained using the new tunes from $$7\,\text {TeV} $$ are shown for the charged-particle and the $$p_\mathrm{T}^\mathrm{sum}$$ densities in the TransMIN, TransMAX, and TransDIF regions, defined as a function of $$p_\mathrm{T}^\mathrm{max}$$. Also shown is the ratio of $$13\,\text {TeV} $$ to $$7\,\text {TeV} $$ results for the five tunes. The TransMIN region increases much more rapidly with energy than the TransDIF region. For example, when using CUETP$$8$$M$$1$$, the charged-particle and the $$p_\mathrm{T}^\mathrm{sum}$$ densities in the TransMIN region for $$5.0<$$$$p_\mathrm{T}^\mathrm{max}$$$$<6.0\,\text {GeV} $$ is predicted to increase by 28 and $$37~\%$$, respectively, while the TransDIF region is predicted to increase by a factor of two less, i.e. by 13 and $$18~\%$$ respectively.

**Fig. 22:**
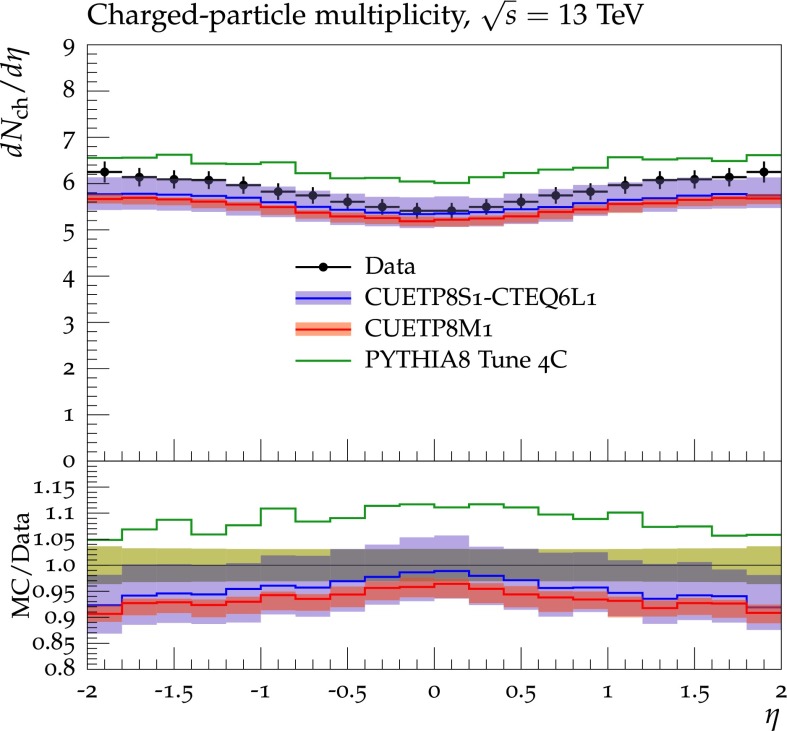
CMS data at $\sqrt{s}=13\,\text {TeV}$ [53] for the charged-particle pseudorapidity distribution, $\mathrm{d}{\mathrm {N}}_{\text {ch}}/\mathrm{d}\eta$, in inelastic proton–proton collisions. The data are compared to predictions of pythia8 using CUETP$8$S$1$-CTEQ$6$L1, CUETP$8$M$1$, and Tune $4$C. The predictions based on CUETP$8$S$1$-CTEQ$6$L1 and CUETP$8$M$1$ are shown with an error band corresponding to the total uncertainty obtained from the eigentunes. Also shown are the ratios of these predictions to the data. The *green band* represents the total experimental uncertainty on the data

In Fig. 22, predictions obtained with pythia8 using CUETP$8$S$1$-CTEQ$6$L1 and CUETP$8$M$1$, and Tune $4$C are compared to the recent CMS data measured at $\sqrt{s} = 13\,\text {TeV}$ [53] on charged-particle multiplicity as a function of pseudorapidity. Predictions from CUETP$8$S$1$-CTEQ$6$L1 and CUETP$8$M$1$ are shown with the error bands corresponding to the uncertainties obtained from the eigentunes. These two new CMS tunes, although obtained from fits to UE data at 7$\,\text {TeV}$, agree well with the MB measurements over the whole pseudorapidity range, while predictions from pythia8 Tune $4$C overestimate the data by about 10 %. This confirms that the collision-energy dependence of the CMS UE tunes parameters can be trusted for predictions of MB observables.

## Summary and conclusions

New tunes of the pythia event generator were constructed for different parton distribution functions using various sets of underlying-event (UE) data. By simultaneously fitting UE data at several center-of-mass energies, models for UE have been tested and their parameters constrained. The improvement in the description of UE data provided by the new CMS tunes at different collision energies gives confidence that they can provide reliable predictions at $\sqrt{s} = 13\,\text {TeV}$, where all the new UE tunes predict similar results for the UE observables.

The observables sensitive to double-parton scattering (DPS) were fitted directly by tuning the MPI parameters. Two $\mathrm {W}$+dijet DPS tunes and two four-jet DPS tunes were constructed to study the dependence of the DPS-sensitive observables on the MPI parameters. The CMS UE tunes perform fairly well in the description of DPS observables, but they do not fit the DPS data as well as the DPS tunes do. On the other hand, the CMS DPS tunes do not fit the UE data as well as the UE tunes. At present, it is not possible to accurately describe both soft and hard MPI within the current pythia and herwig++ frameworks. Fitting DPS-sensitive observables has also provided the DPS effective cross section $\sigma _\mathrm{eff}$ associated to each model. This method can be applied to determine the $\sigma _\mathrm{eff}$ values associated with different MPI models implemented in the current MC event generators for the production of any final-state with two hard particles.

Predictions of pythia8 using the CMS UE tunes agree fairly well with the MB observables in the central region ($|\eta |<2$) and can be interfaced to higher-order and multileg matrix-element generators, such as powheg and MadGraph, while maintaining their good description of the UE. It is not necessary to produce separate tunes for these generators. In addition, we have verified that the measured particle pseudorapidity density at 13$\,\text {TeV}$ is well reproduced by the new CMS UE Tunes. Furthermore, all of the new CMS tunes come with their eigentunes, which can be used to determine the uncertainties associated with the theoretical predictions. These new CMS tunes will play an important role in predicting and analyzing LHC data at 13 and $14\,\text {TeV}$.

## Figures and Tables

**Fig. 23:**
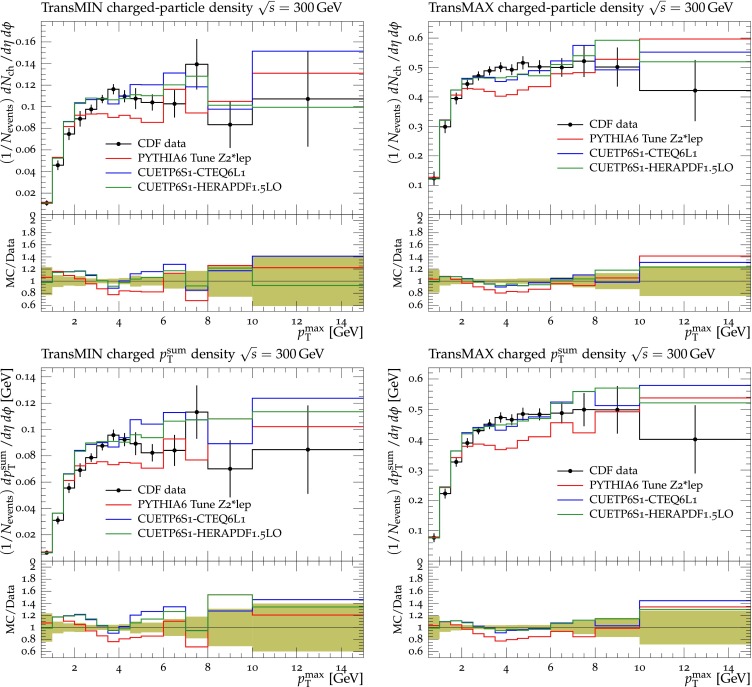
CDF data at $\sqrt{s}=300\,\text {GeV}$ [11] on the particle (*top*) and $p_\mathrm{T}^\mathrm{sum}$ densities (*bottom*) for charged particles with $p_\mathrm{T}\!>\!0.5\,\text {GeV}$ and $|\eta |\!<\!0.8$ in the TransMIN (*left*) and TransMAX (*right*) regions as defined by the leading charged particle, as a function of the transverse momentum of the leading charged-particle $p_\mathrm{T}^\mathrm{max}$. The data are compared to the pythia6 Tune Z$2^*$lep, CUETP$6$S$1$-CTEQ$6$L1 and CUETP$6$S$1$-HERAPDF1.5LO. The *green bands* in the ratios represent the total experimental uncertainties

**Fig. 24:**
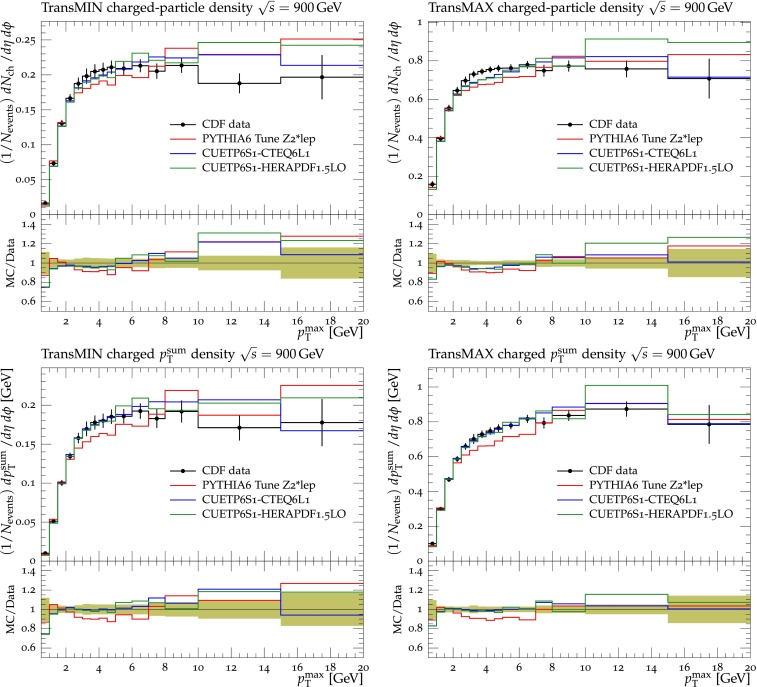
CDF data at $\sqrt{s}=900\,\text {GeV}$ [11] on the particle (*top*) and $p_\mathrm{T}^\mathrm{sum}$ densities (*bottom*) for charged particles with $p_\mathrm{T}\!>\!0.5\,\text {GeV}$ and $|\eta |\!<\!0.8$ in the TransMIN (*left*) and TransMAX (*right*) regions as defined by the leading charged particle, as a function of the transverse momentum of the leading-charged particle $p_\mathrm{T}^\mathrm{max}$. The data are compared to the pythia6 Tune Z$2^*$lep, CUETP$6$S$1$-CTEQ$6$L1 and CUETP$6$S$1$-HERAPDF1.5LO. The *green bands* in the ratios represent the total experimental uncertainties

**Fig. 25:**
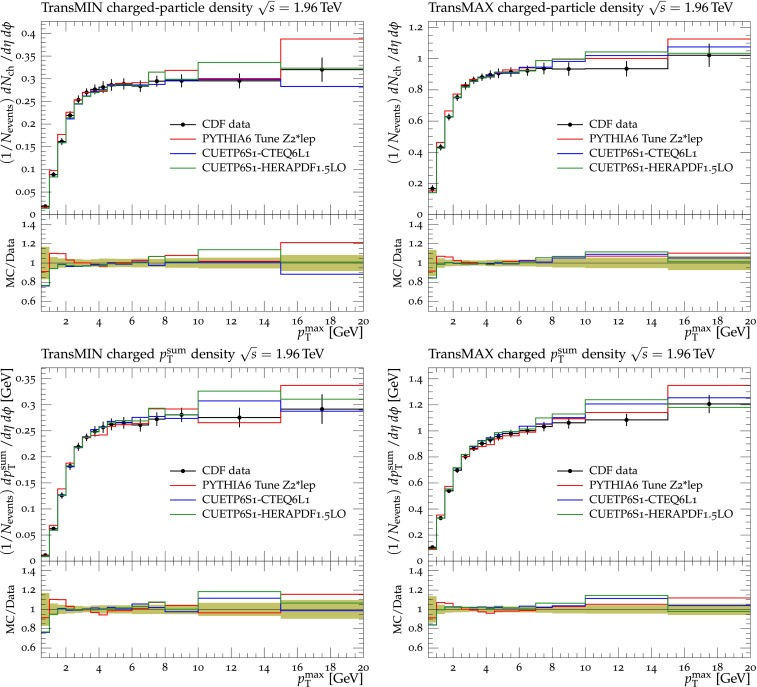
CDF data at $\sqrt{s}=1.96\,\text {TeV}$ [11] on the particle (*top*) and $p_\mathrm{T}^\mathrm{sum}$ densities (*bottom*) for charged particles with $p_\mathrm{T}\!>\!0.5\,\text {GeV}$ and $|\eta |\!<\!0.8$ in the TransMIN (*left*) and TransMAX (*right*) regions as defined by the leading charged particle, as a function of the transverse momentum of the leading charged-particle $p_\mathrm{T}^\mathrm{max}$. The data are compared to the pythia6 Tune Z$2^*$lep, CUETP$6$S$1$-CTEQ$6$L1 and CUETP$6$S$1$-HERAPDF1.5LO. The *green bands* in the ratios represent the total experimental uncertainties

**Fig. 26:**
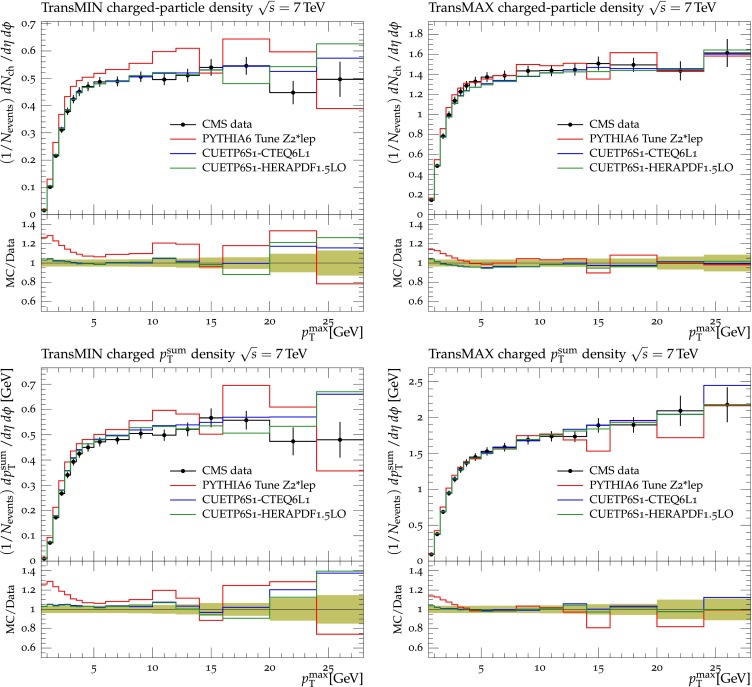
CMS data at $\sqrt{s}=7\,\text {TeV}$ [17] on the particle (*top*) and $p_\mathrm{T}^\mathrm{sum}$ densities (*bottom*) for charged particles with $p_\mathrm{T}\!>\!0.5\,\text {GeV}$ and $|\eta |\!<\!0.8$ in the TransMIN (*left*) and TransMAX (*right*) regions as defined by the leading charged particle, as a function of the transverse momentum of the leading charged-particle $p_\mathrm{T}^\mathrm{max}$. The data are compared to the pythia6 Tune Z$2^*$lep, CUETP$6$S$1$-CTEQ$6$L1 and CUETP$6$S$1$-HERAPDF1.5LO. The *green bands* in the ratios represent the total experimental uncertainties

**Fig. 27:**
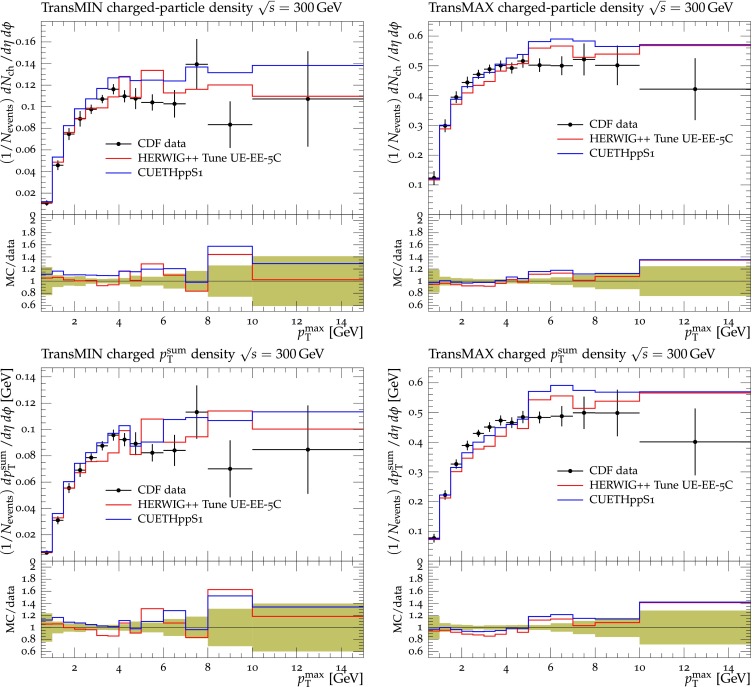
CDF data at $\sqrt{s}=300\,\text {GeV}$ [11] on particle (*top*) and $p_\mathrm{T}^\mathrm{sum}$ densities (*bottom*) for charged particles with $p_\mathrm{T}\!>\!0.5\,\text {GeV}$ and $|\eta |\!<\!0.8$ in the TransMIN (*left*) and TransMAX (*right*) regions as defined by the leading charged particle, as a function of the transverse momentum of the leading charged-particle $p_\mathrm{T}^\mathrm{max}$. The data are compared to the herwig++ Tune UE-EE-$5$C and CUETHppS$1$. The *green bands* in the ratios represent the total experimental uncertainties

**Fig. 28:**
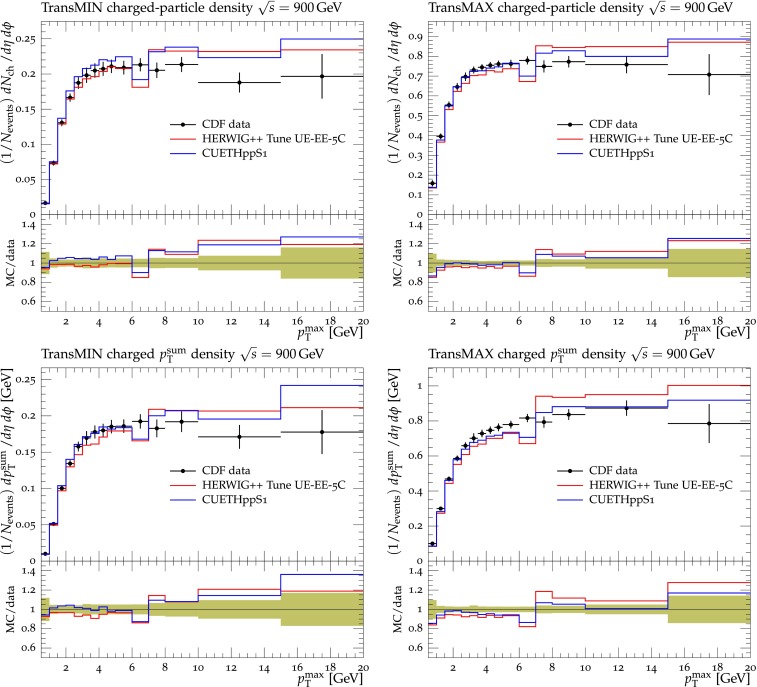
CDF data at $\sqrt{s}=900\,\text {GeV}$ [11] on particle (*top*) and $p_\mathrm{T}^\mathrm{sum}$ densities (*bottom*) for charged particles with $p_\mathrm{T}\!>\!0.5\,\text {GeV}$ and $|\eta |\!<\!0.8$ in the TransMIN (*left*) and TransMAX (*right*) regions as defined by the leading charged particle, as a function of the transverse momentum of the leading charged-particle $p_\mathrm{T}^\mathrm{max}$. The data are compared to the herwig++ Tune UE-EE-$5$C and CUETHppS$1$. The *green bands* in the ratios represent the total experimental uncertainties

**Fig. 29:**
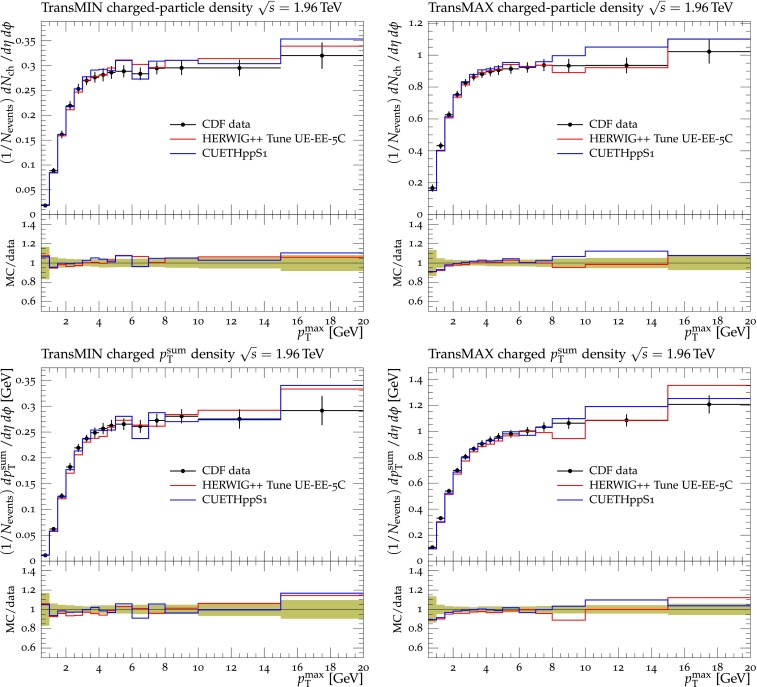
CDF data at $\sqrt{s}=1.96\,\text {TeV}$ [11] on particle (*top*) and $p_\mathrm{T}^\mathrm{sum}$ densities (*bottom*) for charged particles with $p_\mathrm{T}\!>\!0.5\,\text {GeV}$ and $|\eta |\!<\!0.8$ in the TransMIN (*left*) and TransMAX (*right*) regions as defined by the leading charged particle, as a function of the transverse momentum of the leading charged-particle $p_\mathrm{T}^\mathrm{max}$. The data are compared to the herwig++ Tune UE-EE-$5$C and CUETHppS$1$. The *green bands* in the ratios represent the total experimental uncertainties

**Fig. 30:**
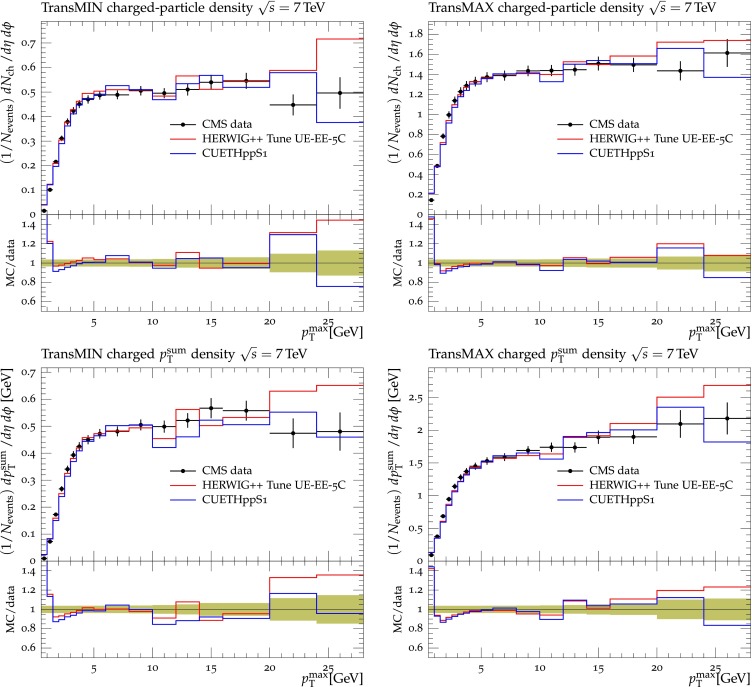
CMS data at $\sqrt{s}=7\,\text {TeV}$ [17] on particle (*top*) and $p_\mathrm{T}^\mathrm{sum}$ densities (*bottom*) for charged particles with $p_\mathrm{T}\!>\!0.5\,\text {GeV}$ and $|\eta |\!<\!0.8$ in the TransMIN (*left*) and TransMAX (*right*) regions as defined by the leading charged particle, as a function of the transverse momentum of the leading charged-particle $p_\mathrm{T}^\mathrm{max}$. The data are compared to the herwig++ Tune UE-EE-$5$C and CUETHppS$1$. The *green bands* in the ratios represent the total experimental uncertainties

**Fig. 31:**
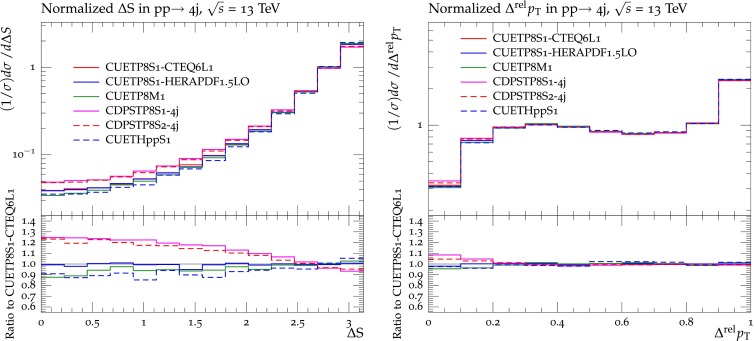
Predictions at $\sqrt{s}=13\,\text {TeV}$ for the normalized distributions of the correlation observables $\varDelta$S (*left*), and $\varDelta ^\mathrm{rel}p_\mathrm{T}$ (*right*) for four-jet production in pp collisions for the three CMS pythia8 UE tunes CUETP$8$S$1$-CTEQ$6$L1, CUETP$8$S$1$-HERAPDF1.5LO, and CUETP$8$M$1$, for CUETHppS$1$, and for CDPSTP$8$S$1$-$4$j and CDPSTP$8$S$2$-$4$j. Also shown are the ratios of the tunes to predictions of CUETP$8$S$1$-CTEQ$6$L1

**Fig. 32:**
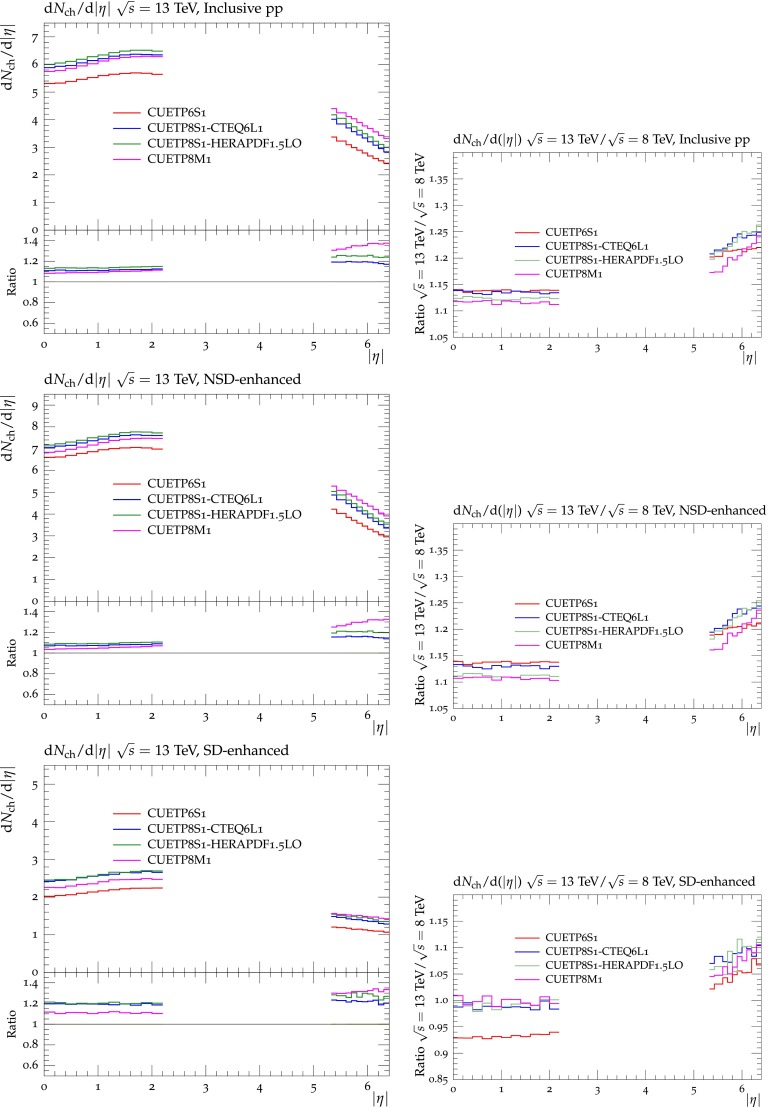
Predictions at $\sqrt{s}=13\,\text {TeV}$ for the charged-particle pseudorapidity distribution $\mathrm{d}{\mathrm {N}}_{\text {ch}}/\mathrm{d}\eta$, for (*top*) inelastic, (*middle*) NSD-enhanced, and (*bottom*) SD-enhanced pp collisions from CUETP$6$S$1$-CTEQ$6$L1, CUETP$8$S$1$-CTEQ$6$L1, CUETP$8$S$1$-HERAPDF1.5LO, and CUETP$8$M$1$. Also shown are the ratios of the tunes to predictions of CUETP$8$M$1$, and the ratio of 13 to $8\,\text {TeV}$ results for each of the tunes (*right*)

## Appendix A: Tables of tune uncertainties

This section provides the values of the parameters corresponding to the eigentunes of the new CMS pythia8 and the herwig++ tunes. A change in the $\chi ^2$ of the fit that equals the absolute $\chi ^2$ value obtained in the tune defines the eigentunes listed in Tables 9, 10, 11 and 12 for the new pythia8 and the new herwig++ tunes. The different parameter values indicated refer to the deviation tunes along each of the maximally independent directions in the parameter space, obtained by using the covariance matrix in the region of the best tune. The number of directions defined in the parameter space equals the number of free parameters *n* used in the fit and results into 2*n* parameter variations, i.e. eigentunes. These variations represent a good set of systematic errors on the given tune.

**Table 9:** Eigentunes sets for CUETP8S1-CTEQ6L1

pythia8 parameter	$1-$	$1+$	$2-$	$2+$	$3-$	$3+$	$4-$	$4+$
MultipartonInteractions:pT0Ref [GeV]	2.101	2.101	2.068	2.135	2.100	2.102	2.079	2.123
MultipartonInteractions:ecmPow	0.191	0.231	0.210	0.211	0.231	0.191	0.191	0.231
MultipartonInteractions:expPow	1.609	1.609	1.602	1.616	1.613	1.605	1.714	1.503
ColourReconnection:range	3.030	3.609	3.313	3.313	3.311	3.314	3.314	3.311

**Table 10:** Eigentunes sets for CUETP8S1-HERAPDF

pythia8 parameter	$1-$	$1+$	$2-$	$2+$	$3-$	$3+$	$4-$	$4+$
MultipartonInteractions:pT0Ref [GeV]	2.000	2.000	1.960	2.043	1.999	2.001	1.968	2.030
MultipartonInteractions:ecmPow	0.275	0.226	0.250	0.250	0.226	0.275	0.274	0.227
MultipartonInteractions:expPow	1.691	1.690	1.681	1.700	1.695	1.686	1.831	1.559
ColourReconnection:range	6.224	5.972	6.096	6.096	6.101	6.091	6.091	6.101

**Table 11:** Eigentunes sets for CUETP8M1

pythia8 parameter	$1-$	$1+$	$2-$	$2+$
MultipartonInteractions:pT0Ref [GeV]	2.403	2.402	2.400	2.405
MultipartonInteractions:ecmPow	0.253	0.251	0.253	0.252

**Table 12:** Eigentunes sets for CUETHppS1

herwig++ parameter	$1-$	$1+$	$2-$	$2+$	$3-$	$3+$	$4-$	$4+$
MPIHandler:InvRadius	2.290	2.227	2.318	2.196	2.272	2.237	2.254	2.256
RemnantDecayer:colourDisrupt	0.396	0.811	0.634	0.623	0.632	0.625	0.596	0.666
MPIHandler:Power	0.396	0.351	0.331	0.408	0.399	0.342	0.361	0.381
ColourReconnector:ReconnectionProbability	0.615	0.460	0.529	0.527	0.523	0.533	0.444	0.626

## Appendix B: Comparisons of PYTHIA6 UE tunes to data

Figures 23, 24, 25 and 26 show the CDF data at $\sqrt{s} = 0.3$, 0.9, and $1.96\,\text {TeV}$, and the CMS data at $\sqrt{s} = 7\,\text {TeV}$ on charged-particle and $p_\mathrm{T}^\mathrm{sum}$ densities in the TransMIN and TransMAX regions, as a function of the transverse momentum of the leading charged-particle $p_\mathrm{T}^\mathrm{max}$. The distributions are compared to predictions obtained with pythia6 Tune Z$2^*$lep and the two new CUETP$6$S$1$-CTEQ$6$L1 and CUETP$6$S$1$-HERAPDF1.5LO. The new CMS pythia6 tunes are able to describe the measurements better than Tune Z$2^*$lep, in both the rising and the plateau regions of the spectra.

## Appendix C: Comparisons to HERWIG++ UE tunes to data

Figures 27, 28, 29 and 30 show the CDF data at $\sqrt{s} = 0.3$, 0.9, and $1.96\,\text {TeV}$, and the CMS data at $\sqrt{s} = 7\,\text {TeV}$ on the charged-particle and $p_\mathrm{T}^\mathrm{sum}$ densities in the TransMIN and TransMAX regions as a function of $p_\mathrm{T}^\mathrm{max}$, and compared with predictions obtained with the herwig++ Tune UE-EE-5C and the new CUETHppS$1$. These two herwig++ tunes are very similar and adequately describe the UE data at all four energies.

## Appendix D: Additional comparisons at 13 TeV

In this section, a supplementary collection of comparisons among predictions of the new tunes are shown for DPS and MB observables at 13$\,\text {TeV}$.

### D.1 DPS predictions at 13 TeV

In Fig. 31, the predictions for the DPS-sensitive observables at 13$\,\text {TeV}$ are shown for the three CMS pythia8 UE tunes: CUETP$8$S$1$-CTEQ$6$L1, CUETP$8$S$1$-HERAPDF1.5LO, and CUETP$8$M$1$, for CUETHppS$1$, and for the two CMS pythia8 DPS tunes CDPSTP$8$S$1$-$4$j and CDPSTP$8$S$2$-$4$j. In herwig++, $\sigma _\mathrm{eff}$ is independent of the center-of-mass energy, while pythia8 gives a $\sigma _\mathrm{eff}$ that increases with energy. The pythia8 UE tunes predict that $\sigma _\mathrm{eff}$ will increase by about $7~\%$ between 7 and $13\,\text {TeV}$, while the CDPSTP$8$S$2$-$4$j predicts an increase of about $20~\%$. This results in slightly different predictions for the DPS-sensitive observables at $13\,\text {TeV}$ for the CMS UE tunes and the CMS DPS tunes.

### D.2 MB predictions at 13 TeV

Predictions of the CMS UE tunes at $\sqrt{s}=13\,\text {TeV}$ are shown in Fig. 32 for the charged-particle pseudorapidity distribution, $\mathrm{d}{\mathrm {N}}_{\text {ch}}/\mathrm{d}\eta$, for inelastic, non single-diffraction-enhanced, and single-diffraction-enhanced proton–proton collisions. In Fig. 32, the ratio of 13 to $8\,\text {TeV}$ results is shown for each of the tunes. The densities in the forward region are predicted to increase more rapidly than the central region between 8 and $13\,\text {TeV}$. However, the UE observables in Figs. 20 and 21 increase much faster with center-of-mass energy than do these MB observables.
